# Disruption of the pro-oncogenic c-RAF–PDE8A complex represents a differentiated approach to treating KRAS–c-RAF dependent PDAC

**DOI:** 10.1038/s41598-024-59451-3

**Published:** 2024-04-18

**Authors:** Sean F. Cooke, Thomas A. Wright, Yuan Yan Sin, Jiayue Ling, Elka Kyurkchieva, Nattaporn Phanthaphol, Thomas Mcskimming, Katharine Herbert, Selma Rebus, Andrew V. Biankin, David K. Chang, George S. Baillie, Connor M. Blair

**Affiliations:** 1 College of Medical, Veterinary and Life Sciences, University of Glasgow, Glasgow, Scotland UK; 2 Wolfson Wohl Cancer Research Centre, Institute of Cancer Sciences, University of Glasgow, Glasgow, Scotland UK; 3 Siriraj Centre of Research Excellence for Cancer Immunotherapy, Faculty of Medicine, Siriraj Hospital, Mahidol University, Bangkok, Thailand

**Keywords:** Pancreatic ductal adenocarcinoma, KRAS, c-RAF-PDE8A, Disruptor peptide, Protein–protein interaction, Targeted therapies, Target validation, Cell biology

## Abstract

Pancreatic ductal adenocarcinoma (PDAC) is considered the third leading cause of cancer mortality in the western world, offering advanced stage patients with few viable treatment options. Consequently, there remains an urgent unmet need to develop novel therapeutic strategies that can effectively inhibit pro-oncogenic molecular targets underpinning PDACs pathogenesis and progression. One such target is c-RAF, a downstream effector of RAS that is considered essential for the oncogenic growth and survival of mutant RAS-driven cancers (including KRAS^MT^ PDAC). Herein, we demonstrate how a novel cell-penetrating peptide disruptor (DRx-170) of the c-RAF–PDE8A protein–protein interaction (PPI) represents a differentiated approach to exploiting the c-RAF–cAMP/PKA signaling axes and treating KRAS–c-RAF dependent PDAC. Through disrupting the c-RAF–PDE8A protein complex, DRx-170 promotes the inactivation of c-RAF through an allosteric mechanism, dependent upon inactivating PKA phosphorylation. DRx-170 inhibits cell proliferation, adhesion and migration of a KRAS^MT^ PDAC cell line (PANC1), independent of ERK1/2 activity. Moreover, combining DRx-170 with afatinib significantly enhances PANC1 growth inhibition in both 2D and 3D cellular models. DRx-170 sensitivity appears to correlate with c-RAF dependency. This proof-of-concept study supports the development of DRx-170 as a novel and differentiated strategy for targeting c-RAF activity in KRAS–c-RAF dependent PDAC.

## Introduction

Accounting for > 90% of pancreatic cancers, pancreatic ductal adenocarcinoma (PDAC) is a highly aggressive and treatment resistant malignancy that presents advanced staged patients with a 5 year survival rate of < 5%^1^. The notoriously late onset of symptoms, coupled with the severe lack of reliable biomarkers, often results in patients being diagnosed when the cancer has already metastasised. Consequently, current ‘standard of care’ (SOC: gemcitabine, gemcitabine + nab-paclitaxel, FOLFIRINOX) is often unable to extend median survival rates beyond 1-year^2–4^.

Driven by significant advances in precision medicine, and associated patient biomarker stratification platforms, next-generation targeted therapeutics are overtaking SOC chemotherapeutics in the pancreatic cancer market^2–7^. Central to this are therapies capable of exploiting the KRAS signalosome (i.e., KRAS directly or associated RAS-effector proteins^8,9^), such as the downstream serine/threonine kinase c-RAF^10^. Through its myriad of kinase-dependent and kinase-independent mechanisms, c-RAF regulates cancer cell proliferation, migration, and survival^10–12^. Incidentally, RAS mutant cancers are frequently identified as being c-RAF ‘addicted’, with genetic co-dependencies consistently observed between RAS and c-RAF^10,13^. The significance of this is demonstrated by pre-clinical studies utilising PDX and GEM models of KRAS mutant (KRAS^MT^) pancreatic and lung adenocarcinoma^14–17^. In these models, systemic ablation of c-RAF induced robust tumour regression and was found to synergise with EGFR inhibition. Moreover, acquired resistance (e.g., through the PI3K-AKT-mTOR pathway) did not occur and limited toxicity was observed. These findings were attributed specifically to c-RAF’s kinase-independent functions, with RAF-associated kinase activity shown to play a non-essential role in KRAS^MT^ tumour progression^13–17^.

Current clinically approved RAF-targeting therapeutics represent an inadequate approach to treating KRAS^MT^ cancers. Such small molecule inhibitors are limited in their ability to exploit c-RAF beyond its catalytic activity (e.g., ATP active site-directed inhibitor, Sorafenib) or beyond its ability to heterodimerise with B-RAF (e.g., B-RAF ‘dimer breaker’, PLX8394)^10–12, 18–20^. Moreover, the sub-optimal target selectivity of said kinase inhibitors are often associated with adverse side effects and dose-limiting toxicities^18–20^. Consequently, in the context of KRAS^MT^ cancer, c-RAF remains an underexploited drug target (particularly with respect to its kinase-independent functions). Thus, there remains an urgent unmet need for novel approaches to selectively exploiting c-RAF activity.

To this end, we have developed a first in class cell-penetrating disruptor peptide (known also as a ‘mimic’, ‘decoy’ or ‘interference’ peptide) that exploits therapeutically the crosstalk between c-RAF and the cAMP-PKA signaling axes^21–27^. Through disrupting the c-RAF–PDE8A protein–protein interaction (PPI), this peptide (DRx-170) appears to promote the inactivation of c-RAF via an allosteric mechanism that is dependent upon inhibitory PKA phosphorylation. Targeted disruption of c-RAF–PDE8A suppressed the proliferation, adhesion, and migration of a human KRAS^MT^ PDAC cell line, independent of MAPK signaling. Consistent with previous reports highlighting the synergism of combined c-RAF/EGFR inhibition, DRx-170’s anti-cancer activity was enhanced when combined with the irreversible EGFR/ERBB family inhibitor afatinib. Our findings not only highlight a pro-oncogenic role for the c-RAF–PDE8A protein complex in RAS-RAF driven PDAC, but further demonstrate a differentiated approach to exploiting c-RAF activity through DRx-170.

## Results

### DRx-170 selectively binds a novel non-active site on c-RAF’s kinase domain

**Figure 1:**
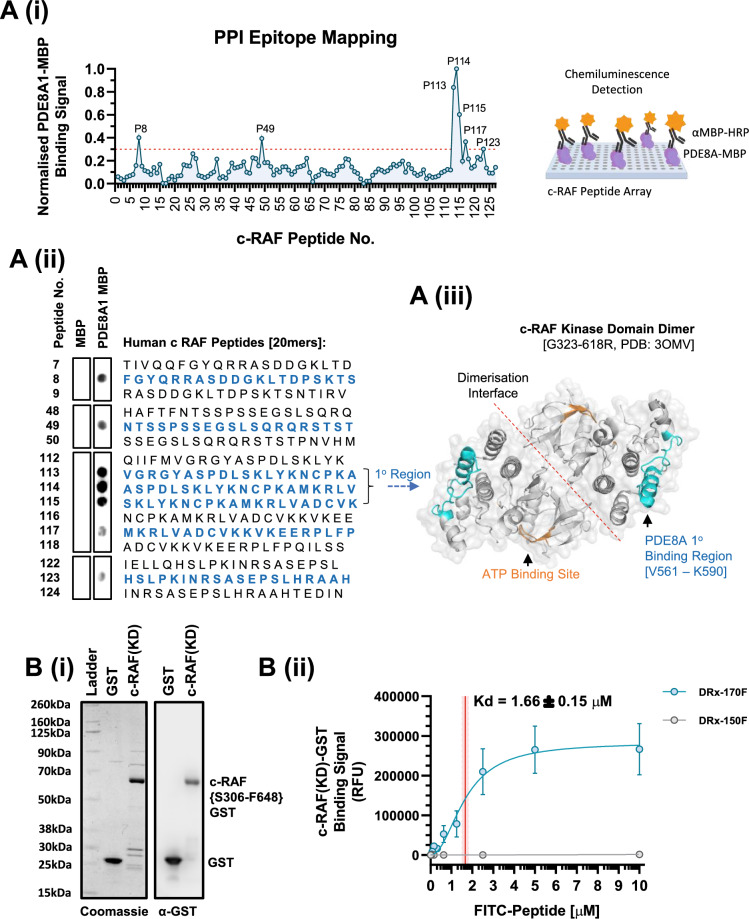
Disruptor peptide directly binds c-RAF. (**A**)(i) Peptide profile mapping the c-RAF epitopes in which human recombinant PDE8A1-MBP^26^ binds, with graphical representation of assay to right of graph. (**A**)(ii) Representative human c-RAF peptide array (20mers) highlighting primary and secondary bind regions and (**A**)(iii) proposed PDE8A1 primary binding region superimposed onto a 3D c-RAF kinase domain structure (PDB: 3OMV^28^), located at a site within c-RAF’s C-Lobe and removed from the active ATP binding site and dimerisation interface. (**B**)(i) Representative coomassie and immunoblot of GST and catalytically ‘active’ c-RAF kinase domain (KD: S306-F648, Y340D/Y341D)–GST proteins. (**B**)(ii) DRx-170F disruptor peptide (blue, N = 5), but not DRx-150F negative control peptide (grey, N = 4), directly binds c-RAF(KD)-GST protein (MEAN ± SEM, Kd = 1.66 ± 0.15 µM). *KD* kinase domain.

Following the discovery of the c-RAF–PDE8A PPI^26^, where c-RAF was shown to bind PDE8A within a conserved region upstream of its catalytic domain (PDE8A1: N442–I476), we sought to conversely characterise the c-RAF epitope(s) in which PDE8A binds (Fig. 1A). Epitope mapping was carried out utilising peptide array screening, where full-length human PDE8A1-MBP was shown to bind human c-RAF (20mer) peptides: 8 (F36–S55), 49 (N241–T260), 113-115 (V561–K590), 117 (M581–P600) and 123 (H611–H630) (Fig. 1A). MBP only control did not bind c-RAF peptides, indicative of specific PDE8A1 binding. The resulting PDE8A1 binding signal was normalised as a fraction of the strongest binding peptide (i.e., peptide 114, A566–V585, normalised binding signal = 1, Fig. 1A(i)). Subsequently, peptides 113-115 were denoted as the primary binding region (normalised binding signal: > 0.5), whilst peptides 8, 49, 117 and 123 were considered secondary (normalised binding signal: 0.3–0.5). Superimposing the primary binding region onto an already solved crystal structure of c-RAF (PDB: 3OMV, Fig. 1A(iii)^28^) suggested that PDE8A1 interacts with c-RAF at a novel non-active site on the C-lobe of its kinase domain, removed from its dimerisation interface and consisting mostly of α-helical secondary structure. Additionally, secondary binding sequences included serine residues 43, 259 and 621 (not S233)—all of which are PKA-specific phosphorylation sites known to directly promote c-RAF inactivation through (i) dissociation from upstream membrane-bound RAS (i.e., pS43) and (ii) 14-3-3 recruitment, binding, and consequential conformational closure (i.e., pS233, pS259 and pS621)^29–39^.

Due to the disordered nature of RAF kinase termini, purification and generation of full-length RAF proteins are highly challenging. Thus, catalytically ‘active’ c-RAF kinase domain (‘KD’) protein was utilised to determine target engagement between c-RAF and DRx-170; a novel cyclic cell-penetrating c-RAF–PDE8A disruptor peptide derived from the minimum binding domain on PDE8A1 in which c-RAF binds (i.e., R^454^RLSGNEYVLST^465^)^23,26^. GST-fusion human c-RAF kinase domain protein was co-incubated with increasing concentrations [0.0006–10 µM] of (i) FITC-labelled DRx-170 (DRx-170F) or (ii) respective FITC-labelled negative control peptide (DRx-150F) (Fig. 1B). DRx-170F directly bound c-RAF(KD)-GST protein (Kd = 1.66 ± 0.15 µM, Fig. 1B(ii)). DRx-150F did not bind c-RAF(KD)-GST (Fig. 1B(ii)), nor did DRx-170F or DRx-150F bind GST protein control (Fig. S1). Data indicates that DRx-170F selectively binds c-RAF’s kinase domain.

Protein sequence identity analysis (pairwise protein BLAST) was subsequently carried out to determine the level of conservation between the primary and secondary binding sites on c-RAF (Fig S2A), with that of the corresponding B-RAF, A-RAF, KSR1, KSR2, MAP2K1 (MEK1), MAP2K2 (MEK2), MAPK3 (ERK1) and MAPK1 (ERK2) protein sequences. Peptide 8 (F31-S50, Fig. S2B(i)) and 49 (N241-T260, Fig. S2B(ii)) sequence identity was relatively low (0–40%), except for c-RAF’s Q254-T260 region which was highly conserved in A/B-RAF. Sequence identity of peptides 113-117 (V561-P600, Fig. S2B(iii)) and 123 (H611-H630, Fig. S2B(iv)) remained low for KSR1/2, MEK1/2, and ERK1/2 (< 40%). However, sequence identity was considerably higher with A-RAF and B-RAF (72.5–77.5%). Whether key differences in the amino acid composition of c-RAF’s primary and/or secondary binding sites correlate with PDE8A target selectivity/preference (particularly amongst A-RAF, B-RAF and c-RAF) was not investigated in this study. Finally, sequence identity between human c-RAF peptides 8, 49, 113-117 and 123 are highly conserved amongst mice (*Mus musculus*), rats (*Rattus norvegicus*), and dogs (*Canis lupus familiaris*) (Fig. S2C).

### Disruption of the c-RAF-PDE8A complex negatively impacts PDAC cell growth

**Figure 2:**
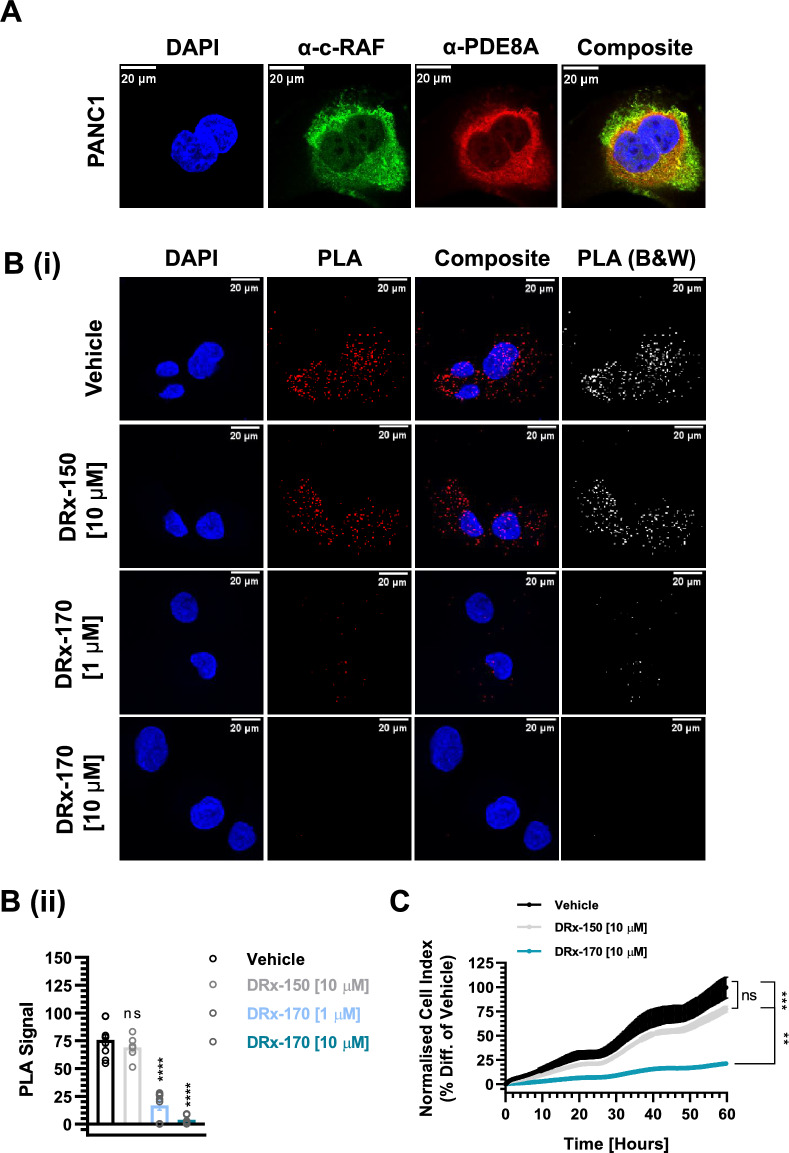
Targeted c-RAF–PDE8A disruption. (**A**) Immunofluorescent co-staining of endogenous c-RAF (mouse α-c-RAF, green) and PDE8A (rabbit α–PDE8A, red) proteins in fixed PANC1 cells. Nuclei counterstained with DAPI (blue) and composite highlighting areas of c-RAF–PDE8A colocalisation (n > 40 cells, scale bar = 20 µm). (**B**)(i), (ii) Proximity ligation assay (PLA) highlighting formation of c-RAF–PDE8A complex (red) within cytoplasm and nuclei of fixed PANC1 cells (scale bar = 20 µm). Disruption of c-RAF–PDE8A complex formation by (4 h) DRx-170, but not DRx-150 or vehicle (1% DMSO)–(ii) shown by dot plot (N = 3, n ≥ 90 cells per sample set). (**C**) RTCA (xCELLigence) analysis demonstrating how c-RAF–PDE8A disruption influences PANC1 cancer cell growth (N = 3). MEAN ± SEM, ns, not significant; **P < 0.01, ***P < 0.001; ****P < 0.0001.

Having already demonstrated a therapeutic role for disrupting the c-RAF–PDE8A PPI in overcoming BRAF inhibitor-resistant (NRAS^MT^) malignant melanoma^22^, we sought to translate our findings in the context of PDAC, where > 90% of patients harbour a KRAS^MT^^2–5^. Following verification of endogenous c-RAF and PDE8A protein expression (Fig. 2A, Fig. S3A), we subsequently confirmed abundant c-RAF–PDE8A complex formation in both the cytoplasm and nuclei of human PANC1 PDAC cell line via PLA (Fig. 2B(i), Fig. S3B). Treatment with DRx-170 significantly downregulated c-RAF–PDE8A complex formation at both [1 µM] and [10 µM] vs. vehicle [1% DMSO] and DRx-150 [10 µM] negative controls (Fig. 2C(i), (ii)). DRx-150 did not significantly influence c-RAF–PDE8A PPI (Fig. 2B(i), (ii)). Nor did the ATP-site directed c-RAF inhibitor, sorafenib (Fig. S3C(i), (ii)). Finally, DRx-170 significantly suppressed relative PANC1 growth vs. vehicle and DRx-150 (Fig. 2C). Thus, DRx-170 readily gains intracellular access, induces targeted disruption of the c-RAF–PDE8A complex and subsequently attenuates PANC1 cell growth.

### Displacing PDE8A upregulates PKA-specific inhibitory c-RAF phosphorylation

**Figure 3:**
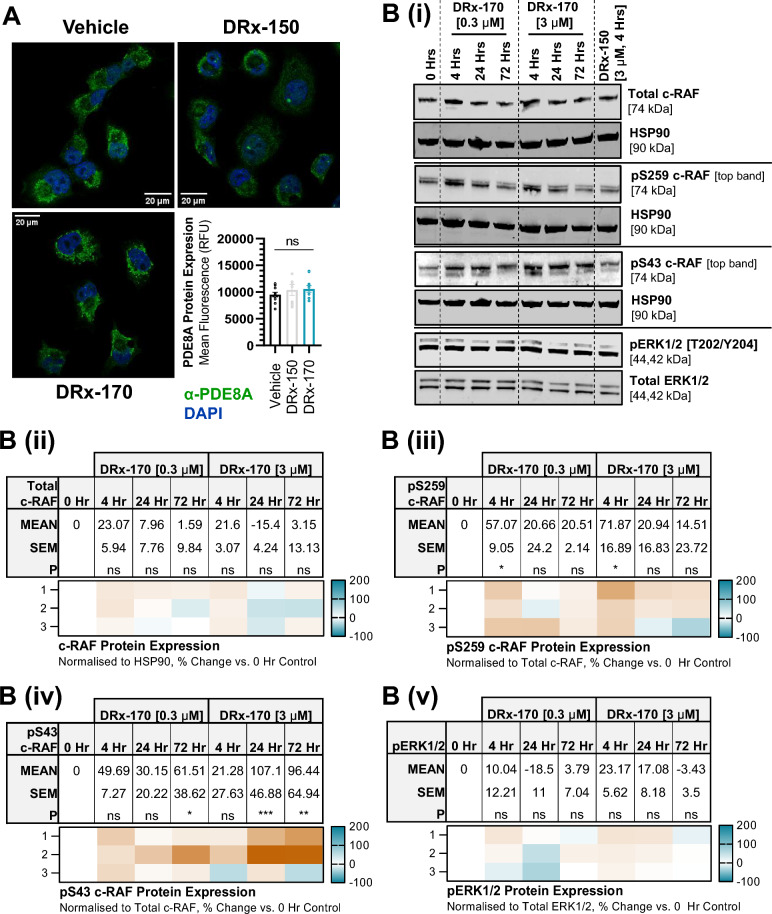
PKA Associated c-RAF Inhibition. (**A**) Immunofluorescent staining of endogenous PDE8A (rabbit α–PDE8A, green) protein in fixed PANC1 cells following 4 h treatment with vehicle (DMSO), DRx-150 (10 µM), or DRx-170 (10 µM). Nuclei counterstained with DAPI (blue) and composite highlighting PDE8A localisation. Respective bar chart of PDE8A protein expression (bottom right, RFU, n ≥ 115 cells per condition, scale bar = 20 µm). (**B**)(i) Representative immunoblots of [1st row] total c-RAF (normalised to HSP90), [2nd row] PKA-specific pS259 c-RAF (normalised to total c-RAF), [3rd row] PKA-specific pS43 c-RAF (normalised to total c-RAF) and [4th row] pT202/pY204 ERK1/2 (normalised to total ERK1/2) protein levels in PANC1 cells. Protein expression at 0 h (N = 3, lane 1) was compared with DRx-170 (4, 24 or 72 h; 0.3 or 3 µM, N = 3, lanes 2–7), DRx-150 (4 h, 3 µM, N = 2, lane 8). (**B**)(ii)–(v) Data showing % change in protein expression vs. 0 h time-point represented as MEAN ± SEM (top), with N = 1–3 independent replicates presented as a heatmap (bottom). P, statistical significance; ns, not significant; *P < 0.05; **P < 0.01; ***P < 0.001.

To directly correlate targeted c-RAF–PDE8A disruption with PANC1 growth inhibition, levels of PKA-mediated inhibitory phosphorylation of c-RAF (i.e., phospho-S43 and S259) were measured (Fig. 3). Firstly, total PDE8A protein expression was not significantly altered following treatment with [10 µM] DRx-170 (Fig. 3A). Nor was c-RAF protein expression following treatment with [3 µM] DRx-170 (Fig. 3B(i), (ii)). Compared with the 0 h control timepoint (normalised to 0%), DRx-170 significantly upregulated pS259 c-RAF protein levels after 4 h at both [0.3 µM] and [3 µM] (Fig. 3B(i), (iii)). Upregulation did not persist at 24 h or 72 h. Additionally, DRx-170 significantly upregulated pS43 c-RAF protein levels after 72 h at [0.3 µM], and after 24 h and 72 h at [3 µM] (Fig. 3B(i), (iv)). DRx-170 did not alter pERK1/2 levels (Fig. 3B(i), (v)). Lastly, DRx-150 did not significantly influence PDE8A, c-RAF, pS259 c-RAF, pS43 c-RAF or pERK1/2 protein expression (Fig. 3). Thus, through disrupting the c-RAF–PDE8A PPI, DRx-170 facilitates PKA-specific inhibitory phosphorylation of c-RAF serine residues 43 and 259. Phosphorylation of c-RAF at S259 appeared to precede S43.

### Afatinib enhances DRx-170 potency in a 2D and 3D human KRAS^MT^ PDAC model

**Figure 4:**
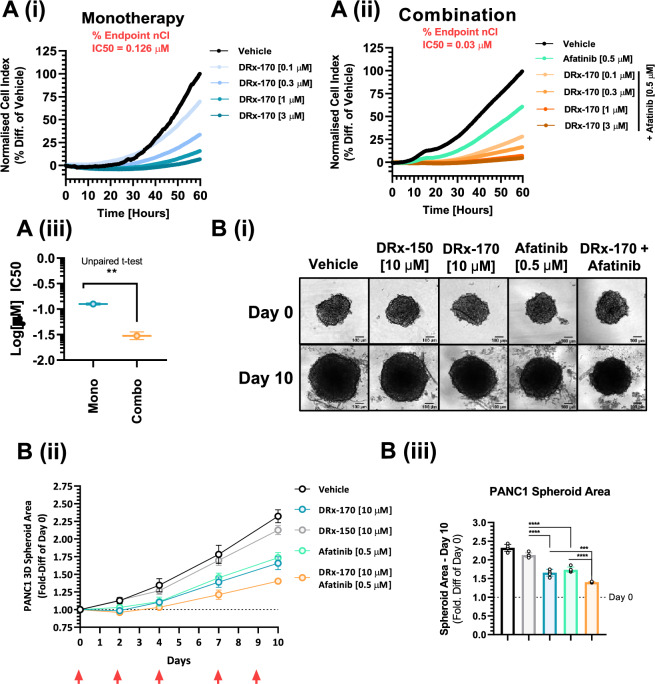
c-RAF–PDE8A disruption suppresses PANC1 growth. (**A**) PANC1 (RTCA) growth following 60 h dose response [0.1–3 µM] with DRx-170 as (**A**)(i) monotherapy and (**A**)(ii) combination with [0.5 µM] afatinib. (**A**)(iii) Respective Log(µM) IC50s (N = 3). (**B**)(i) Day 0 brightfield images (scale bar = 100 µm) of 3D floating PANC1 spheroids and respective Day 10 images following treatment with vehicle [0.2% DMSO], DRx-150, DRx-170, afatinib or DRx-170 + afatinib. (**B**)(ii) Spheroid area over 10 day period and respective bar chart (iii) depicting spheroid area fold-difference vs. Day 0 (n = 5). Red arrows indicate treatment time points. MEAN ± SEM, ns, not significant; **P < 0.01; ****P < 0.0001.

Utilising an existing pancreatic cancer clinical pathology data set, derived from The Human Protein Atlas and The Cancer Genome Atlas (Supplementary Data Set 1), we sought to determine how pancreatic cancer patient survival correlated with EGFR-RAS-RAF mRNA expression. Notably, high EGFR, ERBB2, ERBB3, KRAS, NRAS and c-RAF (but not PDE8A, HRAS, A-RAF or B-RAF) mRNA expression correlated unfavourably with patient survival. Of the limited 176 patients in this data set, 78 (44.3%) expressed high c-RAF mRNA levels. Of these 78 patients, all (100%) expressed high KRAS and/or NRAS mRNA levels. Moreover, 92.3% (72/78) of these patients also expressed high EGFR and/or ERBB2 and/or ERBB3 mRNA levels. Therefore, high c-RAF mRNA levels strongly correlate with KRAS/NRAS and EGFR/ERBB2/ERBB3 mRNA expression. These findings appear consistent with pre-clinical studies supporting a potentially synergistic approach to treating KRAS^MT^ pancreatic adenocarcinomas, through dual inhibition of c-RAF and EGFR^16^. Consequently, we evaluated the combined efficacy of DRx-170 with a 2nd generation (irreversible) EGFR/ERBB-family inhibitor, afatinib (Fig. 4).

Monotherapeutic efficacy of DRx-170 (growth IC50: 0.126 µM, Fig. 4A(i)) vs. PANC1 cells was significantly enhanced (4.2-fold) when combined with [0.5 µM] afatinib (growth IC50: 0.03 µM, Fig. 4A(ii), (iii)). These data were recapitulated in a 3D PANC1 floating spheroid assay, where DRx-170 [10 µM], afatinib [0.5 µM] and respective combination efficacy was assessed over a 10 day treatment period (Fig. 4B). Compared with vehicle [0.2% DMSO] and DRx-150 [10 µM], DRx-170 and afatinib treatment significantly attenuated relative spheroid area (Fig. 4B(i)–(iii)). Combination DRx-170 and afatinib treatment inhibited spheroid area significantly more than respective monotherapies. These results further demonstrate a therapeutic role for dual inhibition of c-RAF and EGFR in KRAS^MT^ PDAC.

Noticeable differences in DRx-170 potency were observed in 2D vs. 3D PANC1 cellular models. Consequently, we sought to determine if this was attributed to DRx-170’s duration of activity and/or stability (Fig. S4). Firstly, DRx-170 activity was assessed in the context of low (2%) and high (10%) FBS vs. 2D PANC1 cells (Fig. S4A). No significant difference in PANC1 growth inhibition was observed (Fig. S4A). However, DRx-170’s ability to suppress the relative rate of PANC1 growth (i.e., % slope of nCI curve) significantly reduced after initial 24 h treatment period; with PANC1 growth accelerating ~ 24 h post-treatment (Fig. S4A). In addition to relative serum stability, DRx-170 appeared stable in both (in vitro) rat plasma (T_½_ > 180 min) and rat liver microsomes (estimated Clint < 13.2 µL/min/mg protein) (Fig. S4B). Moreover, DRx-170 activity was highly comparable regardless of whether it was synthesised as a TFA, chloride or acetate counterion (Fig. S4C). These findings demonstrate that, in these in vitro contexts, DRx-170 stability (and therefore activity) persists for up to 24 h. Whether increased treatment frequency (e.g., every day; q.d.) would enhance 3D PANC1 spheroid growth inhibition was not assessed in this study.

### DRx-170 attenuates PDAC cell adhesion and migration

**Figure 5:**
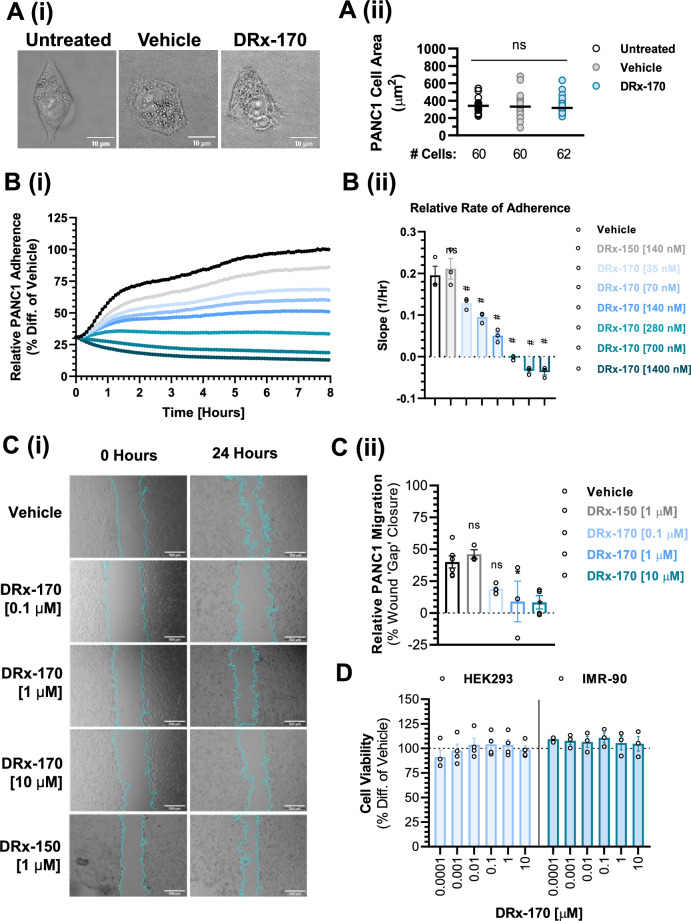
c-RAF–PDE8A disruption attenuates PANC1 adherence and migration. (**A**) Cell area (µm^2^) in untreated vs. DMSO vs. DRx-170 (1 μM) treated (24 h) PANC1 cells (n ≥ 60 cells per group, N = 3, scale bar = 10 µm). (**B**)(i) RTCA (xCELLigence) of PANC1 cell adherence following 8 h treatment with vehicle (1% DMSO), DRx-150 (0.14 µM) or DRx-170 (0.035–1.4 µM). (**B**)(ii) Representative bar chart of relative PANC1 (slope of curve) adherence (N = 3). ns, not significant; #, P < 0.05 vs. DRx-150 and Vehicle. (**C**)(i) PANC1 cell migration analysis (in vitro wound healing, scale bar = 500 µm) following 24 treatment with vehicle (1% DMSO), DRx-150 (1 µM), DRx-170 (0.1–10 µM). Blue outline highlights ‘wound’ at time point 0 h and 24 h. (**C**)(ii) Representative bar chart of relative PANC1 migration (i.e., relative % wound gap closure) (N ≥ 3). (**D**) In vitro cell viability (endpoint) assessment of non-cancerous human cell lines HEK293, IMR-90 following 72 treatment with DRx-170 (0.001–10 µM, N ≥ 3). Horizontal line represents vehicle 100% viability control MEAN ± SEM, ns, not significant; *P < 0.05.

RTCA xCELLigence technology measures a combination of cell proliferation, adhesion, and cell size. To further investigate the observable differences in 2D vs. 3D potency (Fig. 4.), we assessed DRx-170’s influence on PANC1 cell morphology and adhesion (Fig. 5). Firstly, through measuring individual cell area, DRx-170 [1 µM] was shown to have no effect on PANC1 cell size (Fig. 5A). PANC1 adherence was then measured using the RTCA xCELLigence platform. PANC1 cells adhere rapidly (approximately 4–6 h^40^) and their population doubling time is slow (approximately 52 h, ATCC: CRL-1469). Thus, initial increases in cell index (measured via RTCA xCELLigence platform) are largely indicative of cellular adherence, not proliferation. Resultingly, relative PANC1 adherence was assessed over an 8 h period following simultaneous cell seeding and treatment (Fig. 5B). DRx-170 induced potent inhibition of relative PANC1 adhesion (Fig. 5B). DRx-150 did not negatively impact adhesion (Fig. 5B). In addition to the already observed anti-proliferative actions of DRx-170 (Fig. 4), these results demonstrate DRx-170’s ability to also inhibit PANC1 cell adhesion. Therefore, differences in DRx-170 activity in 2D vs. 3D PANC1 cell models is likely attributed to its additional anti-adhesive activities, and not a change in cell size.

Out with this study, PDE8A and c-RAF have been shown to play an important role in regulating cell adhesion and migration^39,41–44^. Resultingly, we investigated DRx-170’s ability to influence PANC1 cell migration utilising an in vitro wound healing assay (Fig. 5C). DRx-170, but not DRx-150, significantly suppressed PANC1 cell migration over a 24 h treatment period (Fig. 5C). These findings demonstrate how DRx-170 mediated disruption of the c-RAF–PDE8A PPI not only attenuates PANC1 cell proliferation, but also PANC1 cell adhesion and migration. DRx-170’s anti-proliferative activity does not extend to non-cancerous HEK293 (epithelial) or IMR-90 (fibroblast) human cell lines, as seen by no significant change in relative cell viability (Fig. 5D).

### DRx-170 activity appears to correlate with KRAS–c-RAF dependency

Harnessing publicly available DepMap portal data sets (https://depmap.org/portal/), we interrogated the correlation between RAS-RAF-PDE8A mutational status and c-RAF genetic dependency; determined through genome-scale CRISPR-Cas9 gene essentiality screening (Fig S5, Supplementary Data Set 2). Combined analysis of n = 1079 human cancer cell lines (> 25 lineages) showed that activating mutations in KRAS, NRAS and HRAS correlate significantly with increased c-RAF gene dependency vs. wild-type RAS (Fig. S5A, C–E). No significant difference was observed with PDE8A gene dependency (Fig. S5B). Cancer cell lines harbouring a c-RAF mutation or a non-V600 B-RAF mutation (not an A-RAF mutation), were significantly more dependent upon c-RAF than those that harbour a BRAF V600 mutation (Fig. S5F). Furthermore, PDE8A mutational status does not correlate with c-RAF gene dependency (Fig. S5G). Thus, RAS, c-RAF, and non-V600 B-RAF mutations correlate with increased c-RAF genetic dependency in this data set.

**Figure 6:**
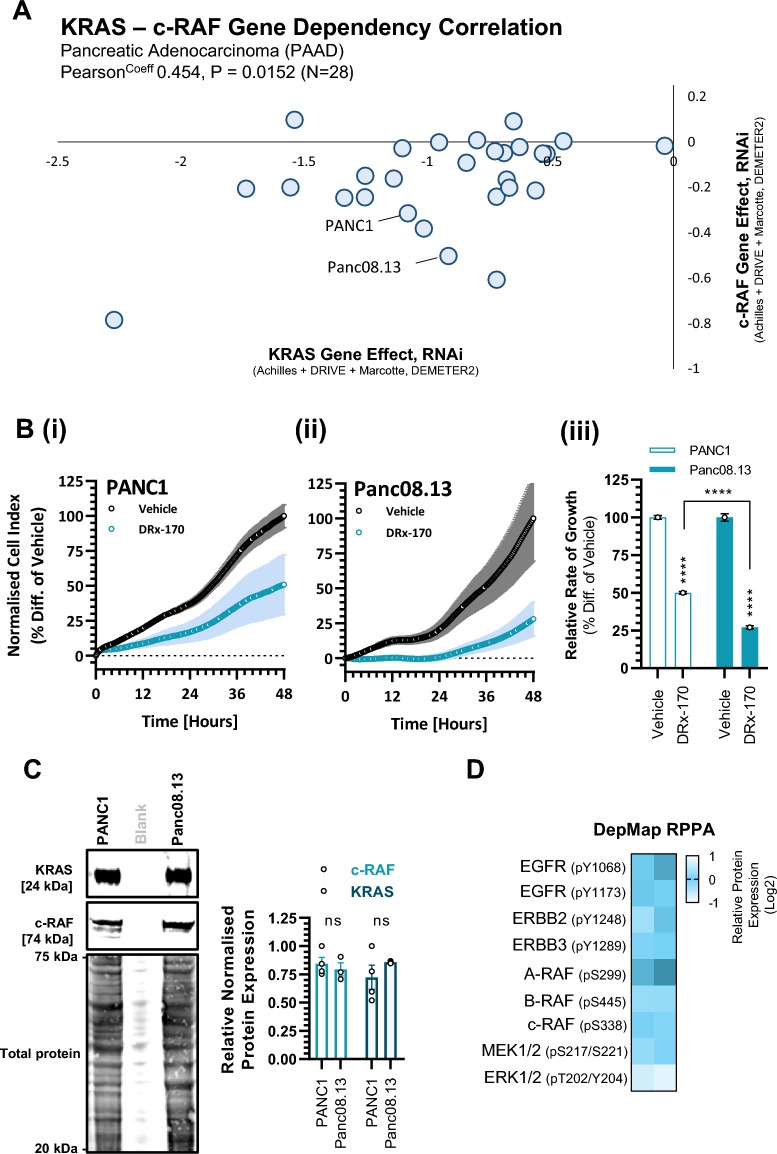
In vitro DRx-170 sensitivity vs. PANC1 and Panc08.13 human PDAC cell lines. (**A**) Scatter plot highlighting KRAS and c-RAF gene effect (RNAi, Achilles + DRIVE + Marcotte, DEMETER2) in 28 pancreatic adenocarcinoma (PAAD) human cancer cell lines (DepMap). See Supplementary Data Set 3*.* (**B**) RTCA (xCELLigence) of (i) PANC1 and (ii) Panc08.13 following 48 h treatment with vehicle [1% DMSO] or DRx-170 [0.1 µM]. (iii) Representative bar chart of relative rate of growth (MEAN ± SEM, n = 6). (**C**) Representative immunoblots of KRAS, c-RAF, Total Protein (Revert^™^ 700 nm stain) from PANC1 and Panc08.13. Corresponding bar chart of relative protein expression (N = 4, ns; not significant, two-way ANOVA). (**D**) DepMap derived Reverse Phase Protein Array (RPPA) data of relative Y1068 EGFR, Y1173 EGFR, Y1248 ERBB2, Y1289 ERBB3, pS299 A-RAF, pS445 B-RAF, pS338 c-RAF, S217/S221 MEK1/2, T202/Y204 ERK1/2 phosphorylated protein expression in PANC1 and Panc08.13 (Log2). See Supplementary Data Set 4*.*

Also correlating with c-RAF genetic dependency is the % prevalence of KRAS^MT^ within specific cancer lineages; with pancreatic adenocarcinoma (PAAD) demonstrating significantly more c-RAF gene dependency than non-small cell lung cancer (NSCLC) and colorectal cancer (CRC) (Fig. S5H). To further explore DRx-170’s potential therapeutic utility in KRAS^MT^ PDAC (beyond PANC1), we assessed DRx-170’s activity against a second human KRAS^MT^ PDAC cell line, Panc08.13. To determine the effect transient knockdown of KRAS or c-RAF has on PANC1 and Panc08.13 cell viability, the DepMap genome-wide RNAi viability screening database (n = 666 human cancer cell lines) was utilised (Fig. 6A, Fig. S6, Supplementary Data Set 3). KRAS gene dependency is similar in PANC1 (−1.078, rank 35/666, Q1) and Panc08.13 (0.914, rank 49/66, Q1) (Fig. S6C). However, Panc08.13 (−0.503, rank 15/666, Q1) is noticeably more dependent upon c-RAF than PANC1 (−0.315, rank 56/666, Q1). Consistent with this, was the observation that Panc08.13 was significantly more sensitive to DRx-170 than PANC1 (Fig. 6B). No significant difference in total KRAS or c-RAF protein expression was observed between PANC1 and Panc08.13 (Fig. 6C). Nor was any marked difference in the relative phosphorylation levels of EGFR-family, RAF-family, MEK or ERK kinases observed (Fig. 6D, Supplementary Data Set 4). Although KRAS GTP hydrolysis levels were not measured in this study, it is worth noting that PANC1 harbours a heterozygous KRAS^G12D^ mutation (allele fraction: 0.667), whilst Panc08.13 is homozygous KRAS^G12D^ (allele fraction: 0.976).

Finally, DRx-170 was evaluated against three additional non-PDAC KRAS^MT^ cancer cell lines (HCT116, NCI-H460, A549) and a RAS-RAF wild-type cancer cell line (U2-OS) (Figs. S7, S8). DRx-170 significantly inhibited cancer cell growth in all KRAS^MT^ cell lines, but not U2-OS RAS-RAF wild-type cells (Figs. S7, S8). Notably, U2-OS is not dependent upon c-RAF (−0.079, rank 278/666, Q2) or KRAS (0.071, rank 606/666, Q3) (Fig. S8).

## Discussion

**Figure 7:**
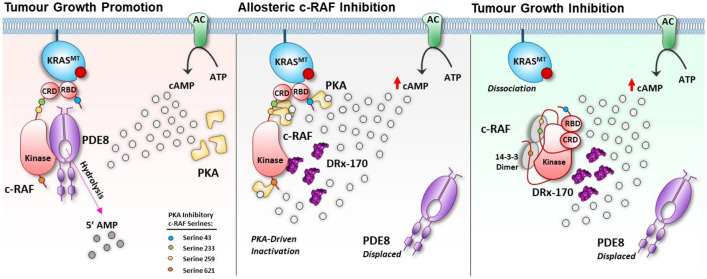
Schematic illustrating how DRx-170 binds c-RAF, displaces PDE8A and exposes c-RAF to surrounding cAMP microenvironment in the context of KRAS^MT^ cancer. De-protection negatively regulates c-RAF activity in a PKA-dependent manner (pS43/pS259 validated, pS233/pS621 untested), promoting c-RAF conformational closure and dissociation from upstream KRAS. This conservative model depicting DRx-170 mechanism of action highlights how DRx-170 attenuates tumourigenesis through facilitating the allosteric inhibition c-RAF. *RBD* ras binding domain; *CRD* cysteine rich domain; *AC* adenylate cyclase; *cAMP* cyclic adenosine monophosphate; *ATP* adenosine triphosphate; *AMP* adenosine monophosphate; *PKA* protein kinase A.

Remodelling of the PPI oncoproteome with highly selective peptide-based disruptor therapeutics represents a rapidly emerging strategy to fine-tuning the disease microenvironment in cancer, significantly de-risking the potential for off-target toxicity^45–49^. This next-generation of targeted therapeutics is making significant progress within the clinical setting (e.g., C/EBPβ dimerisation inhibitor–ST101: NCT04478279^49^) and adds to the growing arsenal of precision medicine tools in oncology research. Given the abysmal survival rate of PDAC, coupled with the severe lack of viable treatment options, novel therapeutic strategies capable of effectively treating this lethal malignancy remain a clear and urgent unmet need. Selectively inhibiting c-RAF’s activity (kinase dependent and independent) represents a persistently attractive therapeutic approach in oncology, offering a high efficacy—low toxicity strategy to treating PDAC and other related RAS^MT^—c-RAF driven malignancies (e.g., lung, colorectal, ovarian, urothelial, and skin cancer). Utilising our first in class cell-penetrating disruptor peptide therapy (DRx-170), we present proof-of-principle data which demonstrates that selective disruption of the pro-oncogenic c-RAF–PDE8A PPI is an encouraging and differentiated approach to inhibiting human KRAS–c-RAF dependent PDAC cell proliferation, adhesion, and migration (Fig. 7).

Through regulating the cAMP microdomain surrounding c-RAF, PDE8A plays a central role in managing the crosstalk between the c-RAF and cAMP-PKA signaling axes^24–26^. Given that c-RAF binds PDE8A at a conserved region upstream of its catalytic domain^26^, it could be suggested that c-RAF can bind all PDE8A1-5 isoforms. Although the functional relevance of this is yet to be discovered, it is highly likely that PDE-specific compartmentalisation plays a significant role in dictating which PDE8A isoforms bind c-RAF, and at which location(s) within the cell this occurs^21,22^. Conversely, protein sequence identity analysis of PDE8A1 binding sites on c-RAF (Fig. 1, Fig. S2) highlight a high degree of sequence conservation within the kinase domain and C-terminus of A-RAF and B-RAF. Though no findings to date have identified PDE8A as a binding partner of A-RAF or B-RAF, our data suggests there is potential for PDE8A to bind all RAF isoforms. Again however, PDE8A isoform compartmentalisation, along with c-RAF–PDE8A localisation and the relative prevalence of RAF homo/heterodimers will presumably direct this. In the context that PDE8A exclusively binds c-RAF, it could be assumed that disrupting the c-RAF–PDE8A PPI with DRx-170 has the potential to regulate all RAF isoforms present within a given RAS-RAF signalosome. Thus, characterising isoform specificity will be a central theme of future research focused on elucidating DRx-170’s mechanism.

As previously documented, disrupting the c-RAF–PDE8A PPI upregulates inhibitory PKA-mediated phosphorylation of c-RAF at serine 259 (S259)^26,39^, a direct indication that c-RAF is in a non-active closed conformation via a 14-3-3 dependent mechanism^33–37^. Following an increase in S259 phosphorylation, upregulation of serine 43 (S43) phosphorylation was observed (Fig. 3). S43 represents another well characterised inhibitory PKA phospho-site on c-RAF, that when phosphorylated stoichiometrically hinders RAS from binding c-RAF’s RBD (Ras Binding Domain)^32–37^. These data suggest (for the first time) that targeted c-RAF–PDE8A disruption promotes RAS-RAF dissociation. Given that RAS-RAF complex formation significantly enhances RAF dimer formation, future studies will look to determine how DRx-170 influences RAF homo/hetero-dimerisation. Moreover, broader profiling of c-RAF’s inhibitory PKA-phospho sites (including S233 and S621) will need carried out to fully characterise their utility as a c-RAF specific biomarker panel consistent with DRx-170 activity. Though it remains a poorly defined area of research, characterisation of corresponding A-RAF (S214, S582) and/or B-RAF (S365, S729) phospho-sites may prove useful in said biomarker panel, and thus remains an area of interest^50–52^.

As anticipated, no change in ERK1/2 activity was observed following c-RAF–PDE8A disruption (Fig. 3). This is in line with pre-clinical studies highlighting a non-essential role for c-RAF kinase-dependent signaling in promoting KRAS^MT^ PDAC^13–17^. Thus, data suggests DRx-170 inhibits KRAS^MT^ PANC1 cell growth, adhesion, and migration independent of c-RAF’s catalytic activity. Research associated with c-RAF’s kinase-independent mechanisms remain within in its infancy. Existing studies have highlighted several c-RAF PPIs that promote cancer cell survival through suppression of pro-apoptotic signaling (e.g., ASK1, MST2, Bcl-2) and regulation of cell motility/de-differentiation (e.g., ROKα)^10–12^. c-RAF’s role in promoting STAT3 activation has also been associated in PDAC and colorectal cancer as being pro-oncogenic, irrespective of c-RAF kinase activity^16,53^. Furthermore, disruption of the recently characterised HSP90-CDC37-c-RAF complex highlights yet another approach to de-stabilising c-RAF^54^. Although these discoveries provide rational direction for future mechanism/biomarker stratification research in RAS^MT^ cancer, it is important to acknowledge both the significant size of the RAF PPI interactome^55–57^, and the fact that c-RAF can promote cancer cell proliferation independent of RAS^53^. Thus, careful elucidation of mechanism(s) is crucial to further differentiating DRx-170 from existing RAF inhibitors.

Precision medicine remains at the forefront of experimental and clinical oncology, supporting the development of targeted therapeutics through accurately characterising each patient’s cancer into specific actionable sub-types. This is also true for PDAC, where the median overall survival of PDAC patients who received genomically matched treatment regime(s) was significantly longer than those who did not (~ 1.71 to 1.96-fold longer)^5,58^. Of relevance, PDAC biomarker signature studies have robustly demonstrated a correlation with DNA-damage response, replication stress, and receptor tyrosine kinase enrichment with platinum-based/PARP inhibitors, cell-cycle inhibitors, and EGFR/ERBB-family inhibitors respectively^59,60^. Thus, to foster the successful pre-clinical and clinical development of our c-RAF–PDE8A disruptor therapeutic, it is critical that we clearly categorise biomarkers that: (i) elucidate the mechanism(s) associated with PPI disruption, (ii) allow for prediction of treatment sensitive vs. resistant cancer models, and (iii) highlight rational combination therapy strategies that can overcome potential acquired resistance. Our preliminary findings suggest that future investigations should be inclusive of RAS-RAF mutational status (excluding B-RAF V600 mutations) and associated dependencies in the EGFR/ERBB–RAS–RAF signaling pathway.

## Materials and methods

### Antibodies and chemicals

Primary antibodies included ERK1/2 (cell signaling, 4696), pERK1/2 (cell signaling, 9101), PDE8A (protein-tech, 13956–1-AP), c-RAF (cell signaling, 9422), c-RAF (sigma, R2404), c-RAF pS43 (Abcam, ab150365), c-RAF pS259 (cell signaling, 9421), KRAS (protein tech, 12063–1-AP), HSP90 (Santa Cruz, sc-7947). IRDye (LI-COR) secondary antibodies included 800CW donkey anti-rabbit IgG (926–32213) and 680RD donkey anti-mouse IgG (926–68072). Alexa Fluor secondary antibodies included donkey anti-mouse 647 nm (Thermo, A-31571) and donkey anti-rabbit 488 nm (Thermo, A-48269). Antibodies were diluted in Intercept T20 TBS antibody diluent (LI-COR). Stock concentrations of afatinib dimaleate (2nd generation tyrosine kinase inhibitor, R&D systems, 6812), sorafenib (tyrosine kinase inhibitor, R&D systems, 6814), and all DRx-peptides (Cambridge research biochemicals) were diluted in 100% DMSO to [10 mM]. Compounds were further diluted to ≤ 1% DMSO in PBS or media in all assays. Unless otherwise stated, drug treatments were carried out in appropriate medium containing 2% FBS. DRx-170 is a short sequence (< 3 kDa), cyclic peptide, derived from the previously discovered minimum binding sequence of PDE8A1 in which c-RAF bound (i.e., R^454^RLSGNEYVLST^465^) and conjugated to a polybasic peptide to enhance cell-permeability^26^. DRx-150 is a respective linear negative control peptide, where key binding residues R454, R455, E460 and Y461 were substituted with alanine. DRx-150 and DRx-170 were synthesised by Biosynth (previously Cambridge Research Biochemicals).

### Peptide array epitope mapping

Peptide array experiments were performed by automatic SPOT synthesis as described ^26,27^. Human c-RAF peptides were synthesised onto PEG-derivatized continuous cellulose membrane supports via 9-fluorenylmethyloxycarbonyl chemistry (Fmoc) using the MultiPep 2 Robot (CEM). A far western blot approach was utilised to detect PDE8A1-MBP binding, where-by c-RAF arrays (consisting of 20mer peptide fragments overlapping by five amino acids) were (i) blocked for 1 h at room temperature in 2.5% milk in 1 × TBS, (ii) incubated overnight at 4 °C in [0.1 μM] PDE8A1-MBP (diluted in 1 × TBS, 5% glycerol, pH 7.4), (iii) incubated for 1 h at room temperature in αMBP-HRP primary antibody (1:1000, Abcam: ab49923) and (iv) visualised via ECL detection utilising the C-Digit Blot Scanner (LI-COR). Arrays were washed three times in 1× TBS-T following primary and secondary antibody incubation steps. MBP (i.e., myosin binding protein) alone was used as a negative protein control. PDE8A1-MBP and MBP proteins are described previously^26^.

### Fluorescent ligand-based binding assay

Glutathione coated wells of a pre-blocked, black, clear bottom, 96-well plate (#15340, Thermo) were incubated with 50 ng of c-RAF kinase domain protein (#14-352, Merck) or GST protein (gifted by Prof. George Baillie) and incubated overnight at 4 °C. Wells were then incubated with increasing concentrations of FITC-labelled peptide [0.6–10 μM] for 2 h at room temperature. Excess protein/peptide was removed following each incubation step by washing three times in 1× TBS-T. Protein and peptides were diluted in binding buffer (200 mM NaCl, 50 mM Tris, 5 mM DTT, 5% Glycerol, protease cocktail inhibitor tablet (Roche), pH 7.5). FITC-peptide binding to c-RAF protein was measured using a Tristar 5 multimode microplate reader (Berthold Technologies). Binding affinities were measured via non-linear regression analysis (GraphPad Prism 8.0).

### Cell culture

All cell lines were cultured in media supplemented with 2 mM l-glutamine (v/v), 100 U/I Pen-Strep (v/v) and 10% FBS (v/v) and grown in a humidified environment with 5% CO_2_ at 37 °C. PANC1 (ATCC–CRL-1469), U2-OS (ATCC–HTB-96), IMR-90 (ATCC–CCL-186) and HEK293 (ATCC–CRL-1573) were cultured in complete DMEM. Panc08.13 (ATCC–CRL-2551) complete RPMI with addition of 10 U/mL human recombinant insulin (Sigma). U2-OS cells were gifted from Prof. Karen Vousden’s research group (Francis Crick Institute, London, UK). All other cell lines were purchased from ATCC.

### Immunocytochemistry (ICC)

PANC1 cells were seeded at 0.5 × 10^5^ cells per well of a 12-well plate containing a sterilised 0.13–0.17 mm glass coverslip in complete DMEM and incubated overnight. Cells were fixed in 4% paraformaldehyde (Sigma) for 15 min at room temperature. Cell membranes were permeabilised with 0.1% Triton × 100 (sigma) for 4 min at room temperature. Cells were then blocked for 1 h at room temperature with 10% donkey serum, 1% BSA in PBS. Both PDE8A (rabbit) and c-RAF (mouse) primary antibodies were then diluted 1:100 in 5% donkey serum, 1% BSA in PBS and cells incubated overnight at 4 °C. Secondary Alexa Fluor antibodies were then simultaneously incubated for 1 h at room temperature. Cells were washed three times in PBS between each of the above steps. Finally, coverslips were then mounted onto glass slides with Prolong Gold Antifade Mountant with DAPI (Thermo, P36941). In experiments where PDE8A protein expression was assessed following treatment, PANC1 cells were treated appropriately prior to fixation. PANC1 cells were imaged using a Zeiss (LSM880) confocal microscope.

### In situ proximity ligation assay (PLA)

PANC1 cells were seeded, fixed and permeabilised as per ICC description. Prior to fixation, cells were treated for 4 h with vehicle only (≤ 1% DMSO) or with 1 and/or 10 µM of (i) DRx-170, (ii) DRx-150 or (iii) sorafenib diluted in media containing reduced serum (2% FBS). In situ detection of the endogenous c-RAF–PDE8A PPI was carried out utilising Duolink^®^ proximity ligation assay as per manufacturer’s instructions (Duolink^®^, Merck). Equal concentrations (1:100) of ICC validated c-RAF (mouse) and PDE8A (rabbit) primary antibodies were used in combination with respective Duolink^®^ PLA α-mouse (PLUS) and α-rabbit (MINUS) probes.

### Western immunoblotting

Protein lysates were harvested using lysis buffer (25 mM Tris, 150 mM NaCl, 0.1 mM EDTA, 1% NP-40, 5% glycerol, pH 7.4) supplemented with protease and phosphatase inhibitors (Roche). Protein samples were diluted in SDS sample buffer (10% SDS, 300 mM Tris-HCl, 0.05% bromothymol blue, 10% β-mercaptoethanol) and boiled for 10 min at 70 °C. Proteins were resolved via SDS-PAGE using 4–12% Bis-Tris gels (NuPAGE), transferred to nitrocellulose membranes (GE Healthcare). To allow for simultaneously incubation of more than one antibody with a single membrane, full membranes were cut at appropriate molecular weight markers (see Supplementary ‘Appendix 1.1-All Immunoblots’ for full immunoblots and replicates). Membranes were blocked in Intercept TBS blocking buffer (LI-COR) and incubated overnight in primary antibody at 4 °C. IRDye secondary antibody (1:10,000, LI-COR) was then incubated for 1 h at room temperature and immunoreactive bands visualised using the Odyssey CLx imaging system (LI-COR). Densitometry of immunoreactive bands was carried out using Image J software. All proteins were normalised to their respective housekeeper protein (HSP90) or Revert^™^ 700 Total protein stain (LI-COR, 926-11011). Phosphorylated proteins were normalised to their respective total.

### Real-time cellular analysis xCELLigence assay

Label-free cellular growth of human cancer cell lines were measured using the xCELLigence real-time cellular analysis platform (RTCA, Roche Applied Science) as per manufacturer’s instructions. 96-well E-plates were utilised to measure cellular impedance within each well, providing quantitative measurements associated with cell proliferation, adherence, and morphology (represented as cell index (CI)). To assess the influence of c-RAF–PDE8A disruption on cancer cell growth, cells were seeded at 1 × 10^4^ cells per well. Following complete PANC1 cell adherence (approximately 4–6 h^40^), cells were treated with the appropriate concentration of drug(s) for 24–60 h and CI monitored every 15 min. To assess PANC1 cell adherence, cells were seeded at 2 × 10^4^ cells per well and immediately treated with appropriate concentration of drug for 8 h (CI measured every 5 min). Unless otherwise stated, all treatments were carried out in respective media containing 2% FBS and to a final DMSO concentration of ≤ 1%. CI was normalised to 1 at treatment timepoint and the rate of growth (i.e., slope of CI curve) analysed via linear-regression analyses (GraphPad Prism 8.0).

### 3D-spheroid growth assay

PANC1 cells were seeded at 2 × 10^3^ in wells of a round bottom Nunclon™ Sphera™ 96 well-plate with ultra-low attachment coating (Thermo; #174925), containing 200 μL DMEM (2% FBS, 2 mM l-glutamine, 100 U/I Pen-Strep). To encourage spheroid formation, PANC1 cells were centrifuged at 250 × *g* for 10 min at room temperature. PANC1 spheroids were then allowed to grow for 3 days at 37 °C, 5% CO_2_, humidified air. Spheroids were then treated with appropriate concentration of DRx-peptide/afatinib by first removing 100 μL of media from each well (careful not to disturb spheroid), followed by addition of 100 μL drug at 2× concentration. Cells were treated five times over a 10 day period (day 0, 2, 4, 7 and 9). Spheroids were imaged on day 0, 2, 4, 7 and 10 using a Nikon Eclipse TS2 microscope and spheroid area quantified using Image J software. Data represented as a fold-difference of relative day 0 spheroid measurement (i.e., day 0 normalised to 1).

### PANC1 cell area

PANC1 cells were seeded at 0.5 × 10^5^ cells per well of a 6-well plate in complete DMEM and incubated overnight. PANC1 cells were then treated for 24 h with vehicle (0.5% DMSO), DRx-170 [1 µM] or left untreated. Following treatment, cells were fixed in 4% paraformaldehyde (Sigma) for 15 min at room temperature. Nikon Eclipse TS2 microscope was used to image PANC1 cells and cell area quantified using Image J software.

### In vitro scratch–wound healing assay

PANC1 cells were seeded at 3 × 10^5^ in wells of a 24 well plate and allowed to grow overnight in DMEM (10% FBS, 2 mM l-glutamine, 100 U/I Pen-Strep). Confluent PANC1 monolayer was then manually ‘scratched’ using a sterilised p200 pipette tip. Cells were then washed two times with PBS to remove detached cells. Cells were then incubated in DMEM (2% FBS, 2 mM l-glutamine, 100 U/I Pen-Strep) containing appropriate concentration of DRx-peptide or vehicle (1% DMSO) and denuded area was immediately imaged (brightfield) using a Nikon Eclipse TS2 microscope. ‘Wound’ was imaged at 24 h post-treatment. The area of ‘wound’ was quantified using the ‘Wound Healing Size Tool’ plugin on Image J software and normalised as % gap closure to respective 0 h measurement^61^.

### In vitro cytotoxicity assay

HEK293 and IMR-90 cells were seeded at 5 × 10^3^ in wells of a clear 96-well plate containing complete DMEM (10% FBS, 2 mM l-glutamine, 100 U/I Pen-Strep). Cells were allowed to grow overnight before treatment with DRx-170 [1–10 μM] or Vehicle (1% DMSO) for 72 h. Following treatment, 100 µL of media (total volume/well = 200 µL) was removed from each well and 20 µL of CellTiter 96® aqueous one solution cell proliferation assay (MTS, Promega: G3581) reagent was added. Following 2 h incubation at 37 °C, cell viability was measured as per manufacturer’s instructions (i.e., A490 nm, Tristar 5 multimode microplate reader, Berthold Technologies).

### In vitro microsomal (liver) stability assay

Stability of DRx-170 in rat liver microsomes was carried out (in duplicate, 96-well plate format) externally by Eurofins Integrated Discovery. [10 mM] stock (100% DMSO) of DRx-170 was diluted to [5 µM] (final DMSO concentration of 0.2%) in pre-warmed (37 °C) reaction buffer containing co-factors NADP, G6P and G6PDH before then adding rat liver microsomes. Aliquots were removed at six specific time points (0, 0.08, 0.25, 0.5, 0.75 and 1 h), and quantified via HPLC–MS/MS analysis (utilising a C-18 column). Verapamil was used as a positive control. Non-CYP450 degradation was assessed in samples containing DRx-170 and rat liver microsomes only (no co-factors). DRx-170 remaining at specified time points was quantified by comparing the peak area with the time-point 0 h peak, and resulting data represented as a % difference. Half-life of DRx-170 was determined based on the slope (generated via linear regression analysis) and apparent CLint subsequently calculated.

### In vitro plasma stability assay

Stability of DRx-170 in rat plasma was carried out (in duplicates, 96-well plate format) externally by Eurofins Integrated Discovery. [10 mM] stock (100% DMSO) of DRx-170 was diluted to [10 µM] (final DMSO concentration of 0.1%). Using a thermomixer, rat plasma was pre-warmed to 37 °C. DRx-170 was added to the plasma and aliquots removed at seven specific time points (0, 0.08, 0.25, 0.5, 1, 1.5 and 2 h). Aliquots were transferred to a protein precipitation plate containing acetonitrile, and as per microsomal stability test pulled under vacuum, dried under nitrogen and resuspended (mobile phase) for subsequent LC–MS/MS analysis in C-18 columns (utilising multiple reaction monitoring (MRM)). Bisacodyl was used a positive control. Half-life was calculated as per microsomal stability protocol.

## Statistical analysis

Unless otherwise stated, data were analysed using a one-way or two-way ANOVA test with follow up Dunnett’s or Tukey’s multiple comparison analysis. Data represented as MEAN ± SEM from ≥ 3 replicates were determined significant by a p value < 0.05. All statistical analyses were carried out using GraphPad Prism 8.0 software.

### Supplementary Information

Supplementary Information 1.

Supplementary Figures.

Supplementary Information 2.

Supplementary Information 3.

Supplementary Information 4.

Supplementary Information 5.

## Data Availability

All relevant data needed to determine the conclusions stated within the manuscript are available in the main text or the supplementary materials. Other raw data/materials used in this study are available upon request to corresponding author.

## Abbreviations

PDAC: Pancreatic ductal adenocarcinoma
PAAD: Pancreatic adenocarcinoma
NSCLC: Non-small cell lung cancer
CRC: Colorectal cancer
SOC: Standard of care
MT: Mutant
EGFR/ERBB: Epidermal growth factor receptor family
Η/Κ/NRAS: Rat sarcoma virus protein family
A/B/c-RAF: Rapidly accelerated fibrosarcoma serine/threonine protein kinase family
ERK1/2: Mitogen-activated protein kinase
MEK1/2: Mitogen-activated protein kinase kinase
PDE8A: Phosphodiesterase 8A
PKA: Protein kinase A
cAMP: Cyclic adenosine monophosphate
ATP: Adenosine triphosphate
MBP: Myosin binding protein
GST: Glutathione s-transferase
PLA: Proximity ligase assay
RTCA: Real-time cellular analysis
