# Supplementary material for: Disruption of the pro-oncogenic c-RAF–PDE8A complex represents a differentiated approach to treating KRAS–c-RAF dependent PDAC
Source: Sci Rep. 2024 Apr 18;14:8998. doi: 10.1038/s41598-024-59451-3 (PMC11026450; doi:10.1038/s41598-024-59451-3)
Supplement: Supplementary file 1 — Supplementary Information 1. [file 41598_2024_59451_MOESM1_ESM.pptx]

## Slide 1
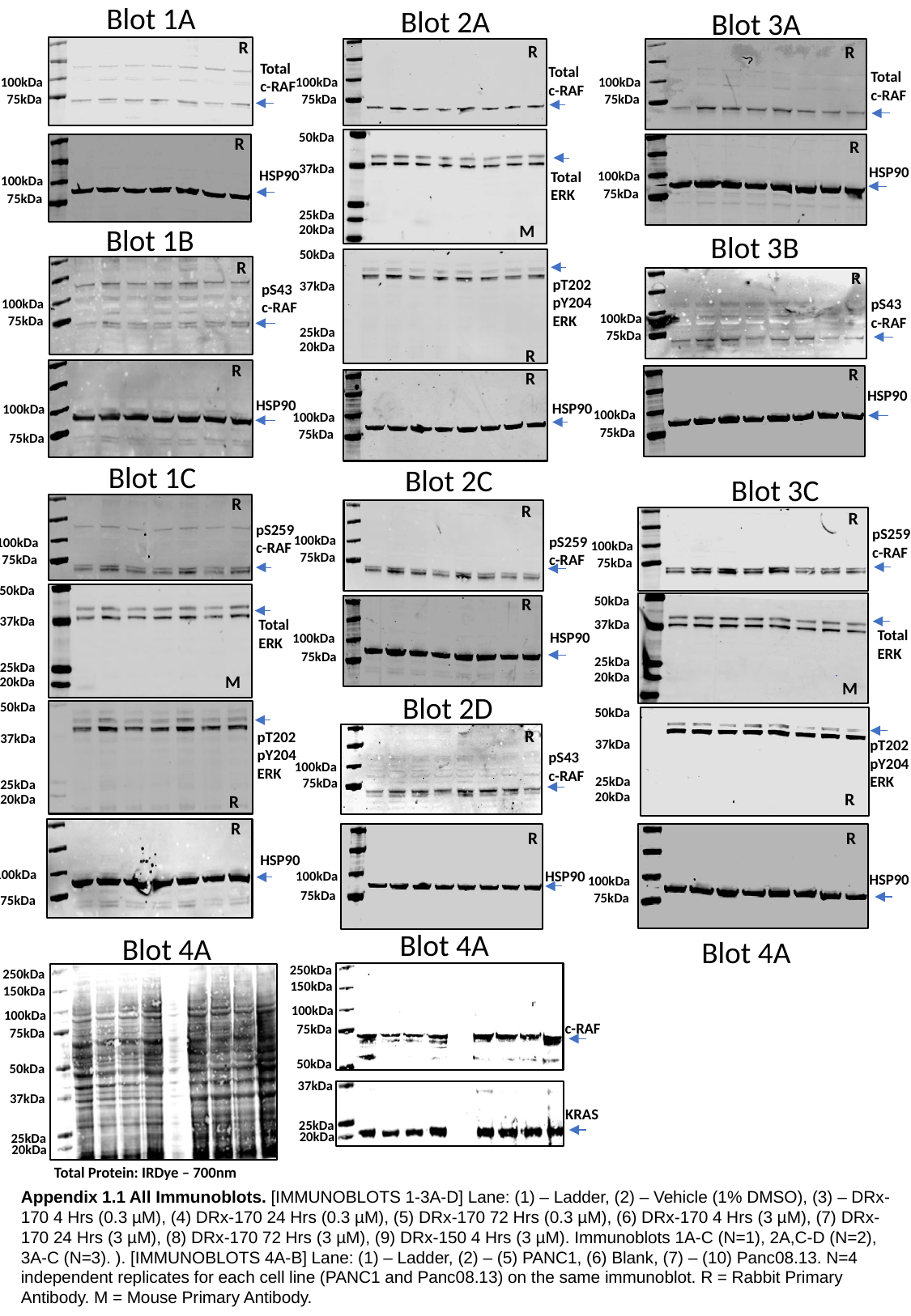

Blot 1A
Blot 2A
Blot 3A
R
R
Total
c-RAF
Total
ERK
M
pT202
pY204
ERK
R
HSP90
R
Total
c-RAF
Total
c-RAF
100kDa
100kDa
100kDa
75kDa
75kDa
75kDa
50kDa
R
R
37kDa
HSP90
HSP90
100kDa
100kDa
75kDa
75kDa
25kDa
Blot 1B
20kDa
Blot 3B
50kDa
R
R
37kDa
pS43
c-RAF
pS43
c-RAF
100kDa
100kDa
75kDa
25kDa
75kDa
20kDa
R
R
R
HSP90
HSP90
100kDa
100kDa
100kDa
75kDa
75kDa
75kDa
Blot 1C
Blot 2C
Blot 3C
R
R
R
pS259
c-RAF
pS259
c-RAF
100kDa
pS259
c-RAF
100kDa
100kDa
75kDa
75kDa
75kDa
50kDa
50kDa
R
37kDa
Total
ERK
37kDa
Total
ERK
HSP90
100kDa
75kDa
25kDa
25kDa
20kDa
M
20kDa
M
Blot 2D
50kDa
50kDa
R
pT202
pY204
ERK
37kDa
pT202
pY204
ERK
37kDa
pS43
c-RAF
100kDa
25kDa
75kDa
25kDa
20kDa
R
R
20kDa
R
R
R
HSP90
100kDa
HSP90
100kDa
HSP90
100kDa
75kDa
75kDa
75kDa
Blot 4A
Blot 4A
Blot 4A
250kDa
250kDa
150kDa
150kDa
100kDa
100kDa
c-RAF
75kDa
75kDa
50kDa
50kDa
37kDa
37kDa
KRAS
25kDa
20kDa
25kDa
20kDa
Total Protein: IRDye – 700nm
Appendix 1.1 All Immunoblots. [IMMUNOBLOTS 1-3A-D] Lane: (1) – Ladder, (2) – Vehicle (1% DMSO), (3) – DRx-170 4 Hrs (0.3 µM), (4) DRx-170 24 Hrs (0.3 µM), (5) DRx-170 72 Hrs (0.3 µM), (6) DRx-170 4 Hrs (3 µM), (7) DRx-170 24 Hrs (3 µM), (8) DRx-170 72 Hrs (3 µM), (9) DRx-150 4 Hrs (3 µM). Immunoblots 1A-C (N=1), 2A,C-D (N=2), 3A-C (N=3). ). [IMMUNOBLOTS 4A-B] Lane: (1) – Ladder, (2) – (5) PANC1, (6) Blank, (7) – (10) Panc08.13. N=4 independent replicates for each cell line (PANC1 and Panc08.13) on the same immunoblot. R = Rabbit Primary Antibody. M = Mouse Primary Antibody.
