# Supplementary material for: Disruption of the pro-oncogenic c-RAF–PDE8A complex represents a differentiated approach to treating KRAS–c-RAF dependent PDAC
Source: Sci Rep. 2024 Apr 18;14:8998. doi: 10.1038/s41598-024-59451-3 (PMC11026450; doi:10.1038/s41598-024-59451-3)
Supplement: Supplementary file 2 — Supplementary Figures. [file 41598_2024_59451_MOESM2_ESM.pptx]

## Slide 1
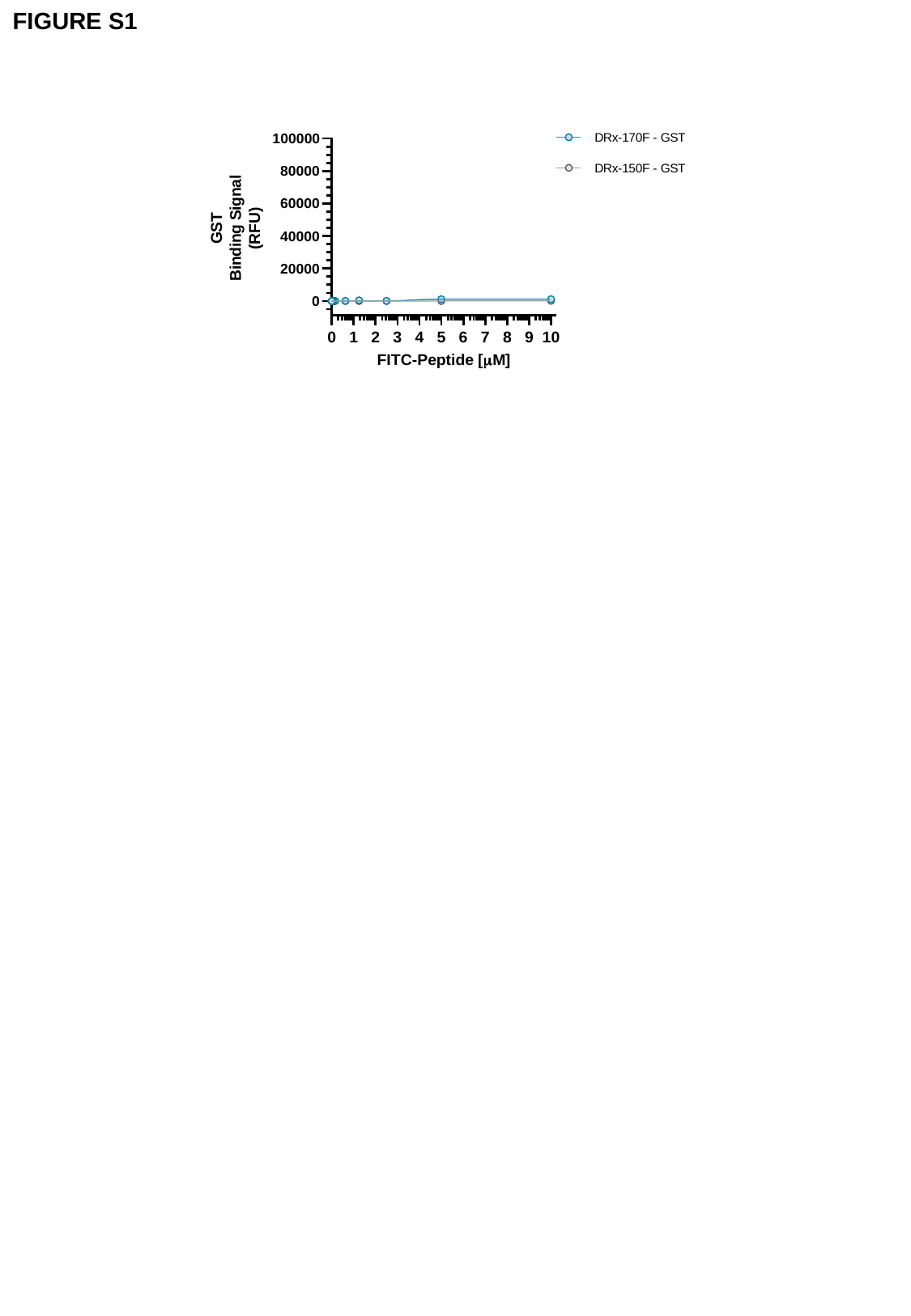

FIGURE S1

## Slide 2
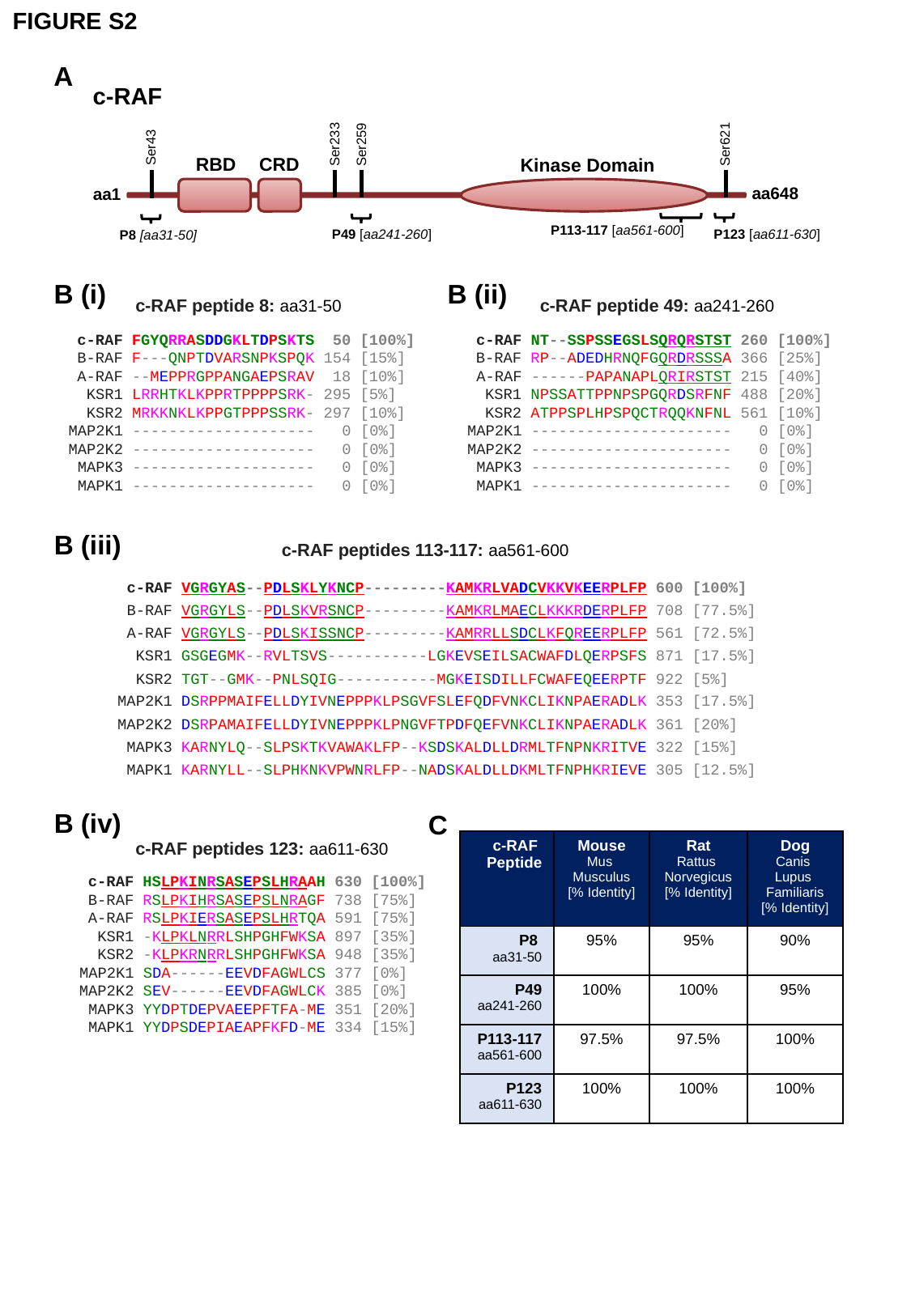

FIGURE S2
A
c-RAF
RBD
CRD
Kinase Domain
aa648
aa1
P113-117 [aa561-600]
P123 [aa611-630]
P49 [aa241-260]
P8 [aa31-50]
Ser233
Ser621
Ser259
Ser43
B (i)
B (ii)
c-RAF peptide 8: aa31-50
c-RAF peptide 49: aa241-260
c-RAF FGYQRRASDDGKLTDPSKTS 50 [100%]
B-RAF F---QNPTDVARSNPKSPQK 154 [15%]
A-RAF --MEPPRGPPANGAEPSRAV 18 [10%]
KSR1 LRRHTKLKPPRTPPPPSRK- 295 [5%]
KSR2 MRKKNKLKPPGTPPPSSRK- 297 [10%]
MAP2K1 -------------------- 0 [0%]
MAP2K2 -------------------- 0 [0%]
MAPK3 -------------------- 0 [0%]
MAPK1 -------------------- 0 [0%]
c-RAF NT--SSPSSEGSLSQRQRSTST 260 [100%]
B-RAF RP--ADEDHRNQFGQRDRSSSA 366 [25%]
A-RAF ------PAPANAPLQRIRSTST 215 [40%]
KSR1 NPSSATTPPNPSPGQRDSRFNF 488 [20%]
 KSR2 ATPPSPLHPSPQCTRQQKNFNL 561 [10%]
MAP2K1 ---------------------- 0 [0%]
MAP2K2 ---------------------- 0 [0%]
MAPK3 ---------------------- 0 [0%]
MAPK1 ---------------------- 0 [0%]
B (iii)
c-RAF peptides 113-117: aa561-600
c-RAF VGRGYAS--PDLSKLYKNCP---------KAMKRLVADCVKKVKEERPLFP 600 [100%]
B-RAF VGRGYLS--PDLSKVRSNCP---------KAMKRLMAECLKKKRDERPLFP 708 [77.5%]
A-RAF VGRGYLS--PDLSKISSNCP---------KAMRRLLSDCLKFQREERPLFP 561 [72.5%]
KSR1 GSGEGMK--RVLTSVS-----------LGKEVSEILSACWAFDLQERPSFS 871 [17.5%]
KSR2 TGT--GMK--PNLSQIG-----------MGKEISDILLFCWAFEQEERPTF 922 [5%]
MAP2K1 DSRPPMAIFELLDYIVNEPPPKLPSGVFSLEFQDFVNKCLIKNPAERADLK 353 [17.5%]
MAP2K2 DSRPAMAIFELLDYIVNEPPPKLPNGVFTPDFQEFVNKCLIKNPAERADLK 361 [20%]
MAPK3 KARNYLQ--SLPSKTKVAWAKLFP--KSDSKALDLLDRMLTFNPNKRITVE 322 [15%]
MAPK1 KARNYLL--SLPHKNKVPWNRLFP--NADSKALDLLDKMLTFNPHKRIEVE 305 [12.5%]
B (iv)
C
| c-RAF Peptide | Mouse Mus Musculus [% Identity] | Rat Rattus Norvegicus [% Identity] | Dog Canis Lupus Familiaris [% Identity] |
| --- | --- | --- | --- |
| P8 aa31-50 | 95% | 95% | 90% |
| P49 aa241-260 | 100% | 100% | 95% |
| P113-117 aa561-600 | 97.5% | 97.5% | 100% |
| P123 aa611-630 | 100% | 100% | 100% |
c-RAF peptides 123: aa611-630
c-RAF HSLPKINRSASEPSLHRAAH 630 [100%]
B-RAF RSLPKIHRSASEPSLNRAGF 738 [75%]
A-RAF RSLPKIERSASEPSLHRTQA 591 [75%]
KSR1 -KLPKLNRRLSHPGHFWKSA 897 [35%]
KSR2 -KLPKRNRRLSHPGHFWKSA 948 [35%]
MAP2K1 SDA------EEVDFAGWLCS 377 [0%]
MAP2K2 SEV------EEVDFAGWLCK 385 [0%]
MAPK3 YYDPTDEPVAEEPFTFA-ME 351 [20%]
MAPK1 YYDPSDEPIAEAPFKFD-ME 334 [15%]

## Slide 3
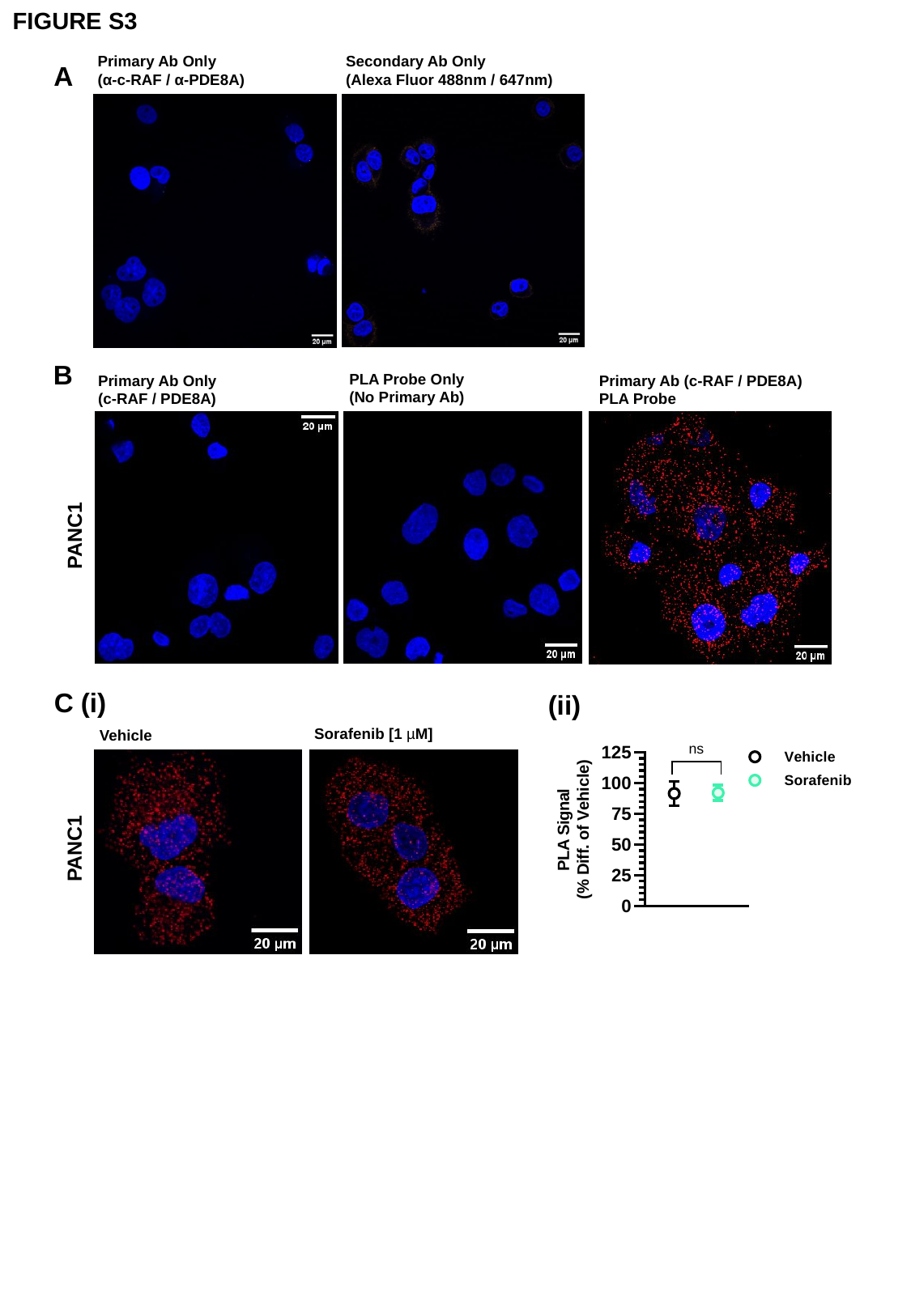

FIGURE S3
Primary Ab Only
(α-c-RAF / α-PDE8A)
Secondary Ab Only
(Alexa Fluor 488nm / 647nm)
A
B
PLA Probe Only
(No Primary Ab)
Primary Ab Only
(c-RAF / PDE8A)
Primary Ab (c-RAF / PDE8A)
PLA Probe
PANC1
C (i)
(ii)
Sorafenib [1 µM]
Vehicle
PANC1

## Slide 4
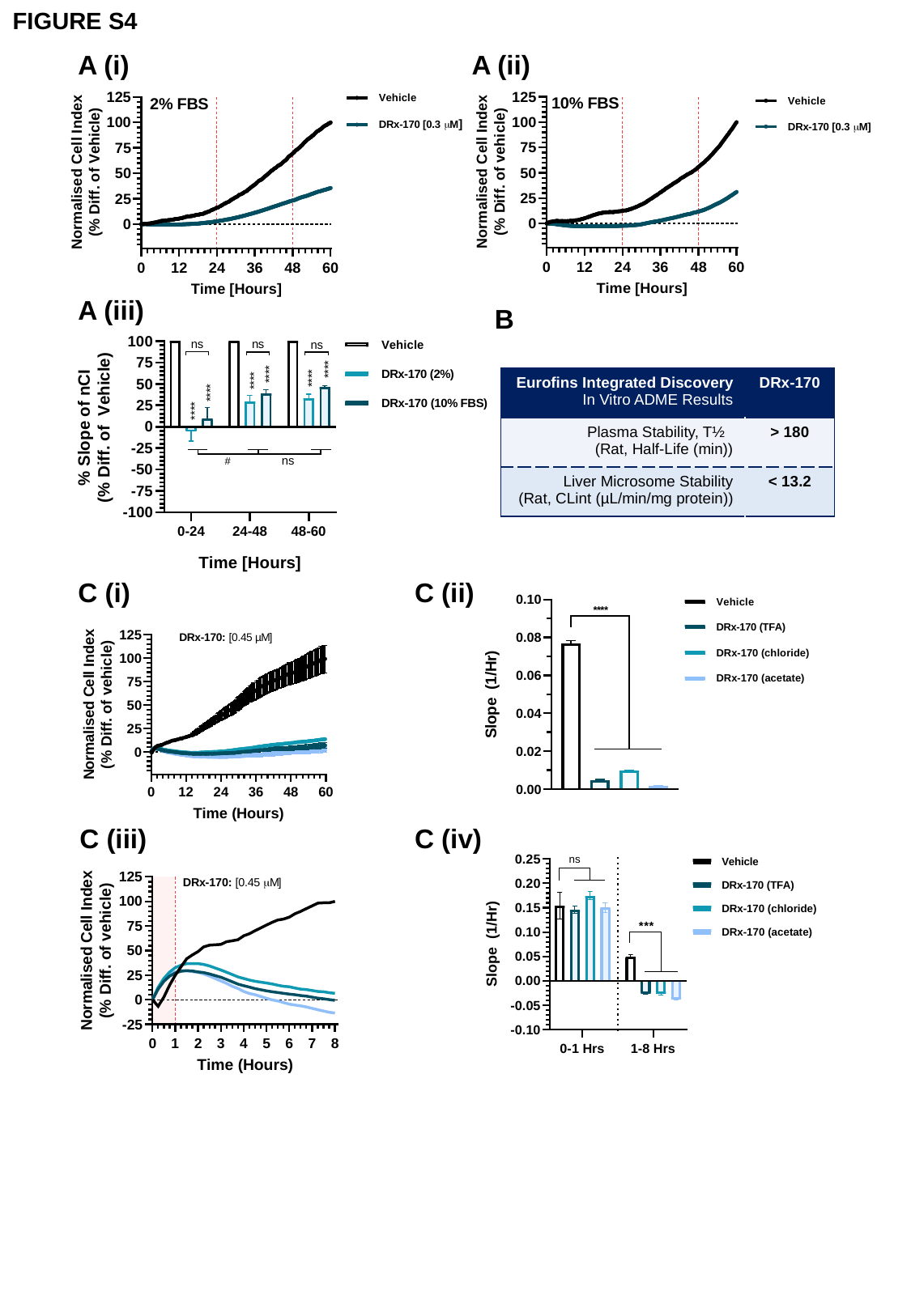

FIGURE S4
A (i)
A (ii)
A (iii)
B
| Eurofins Integrated Discovery In Vitro ADME Results | DRx-170 |
| --- | --- |
| Plasma Stability, T½ (Rat, Half-Life (min)) | > 180 |
| Liver Microsome Stability (Rat, CLint (µL/min/mg protein)) | < 13.2 |
C (i)
C (ii)
C (iv)
C (iii)

## Slide 5
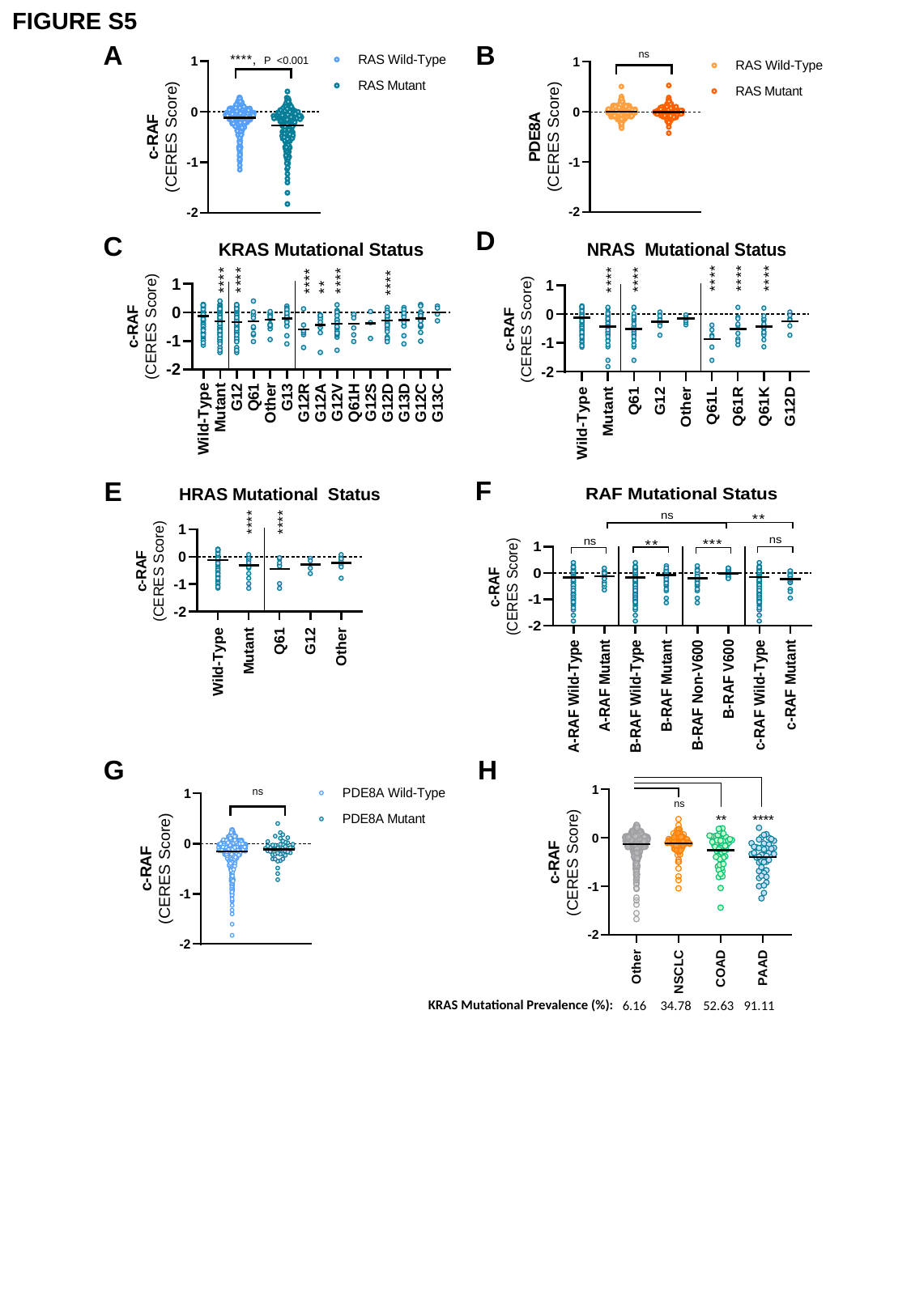

FIGURE S5
A
B
D
C
F
E
G
H
KRAS Mutational Prevalence (%):
6.16
34.78
52.63
91.11

## Slide 6
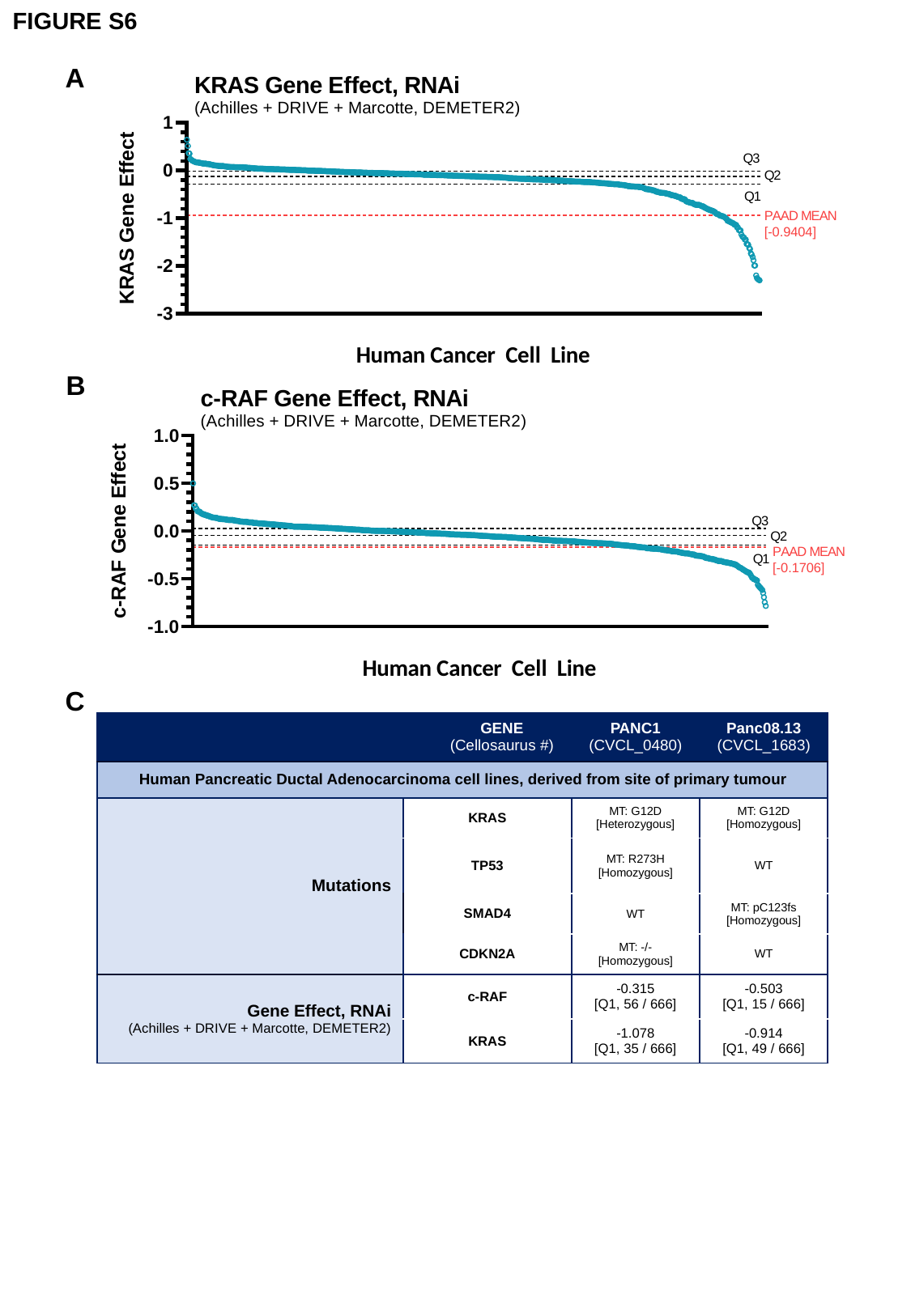

FIGURE S6
A
B
C
| | | GENE (Cellosaurus #) | PANC1 (CVCL\_0480) | Panc08.13 (CVCL\_1683) |
| --- | --- | --- | --- | --- |
| Human Pancreatic Ductal Adenocarcinoma cell lines, derived from site of primary tumour | Lineage | | | |
| Mutations | KRAS | | MT: G12D [Heterozygous] | MT: G12D [Homozygous] |
| | TP53 | | MT: R273H [Homozygous] | WT |
| | SMAD4 | | WT | MT: pC123fs [Homozygous] |
| | CDKN2A | | MT: -/- [Homozygous] | WT |
| Gene Effect, RNAi (Achilles + DRIVE + Marcotte, DEMETER2) | c-RAF | | -0.315 [Q1, 56 / 666] | -0.503 [Q1, 15 / 666] |
| | KRAS | | -1.078 [Q1, 35 / 666] | -0.914 [Q1, 49 / 666] |

## Slide 7
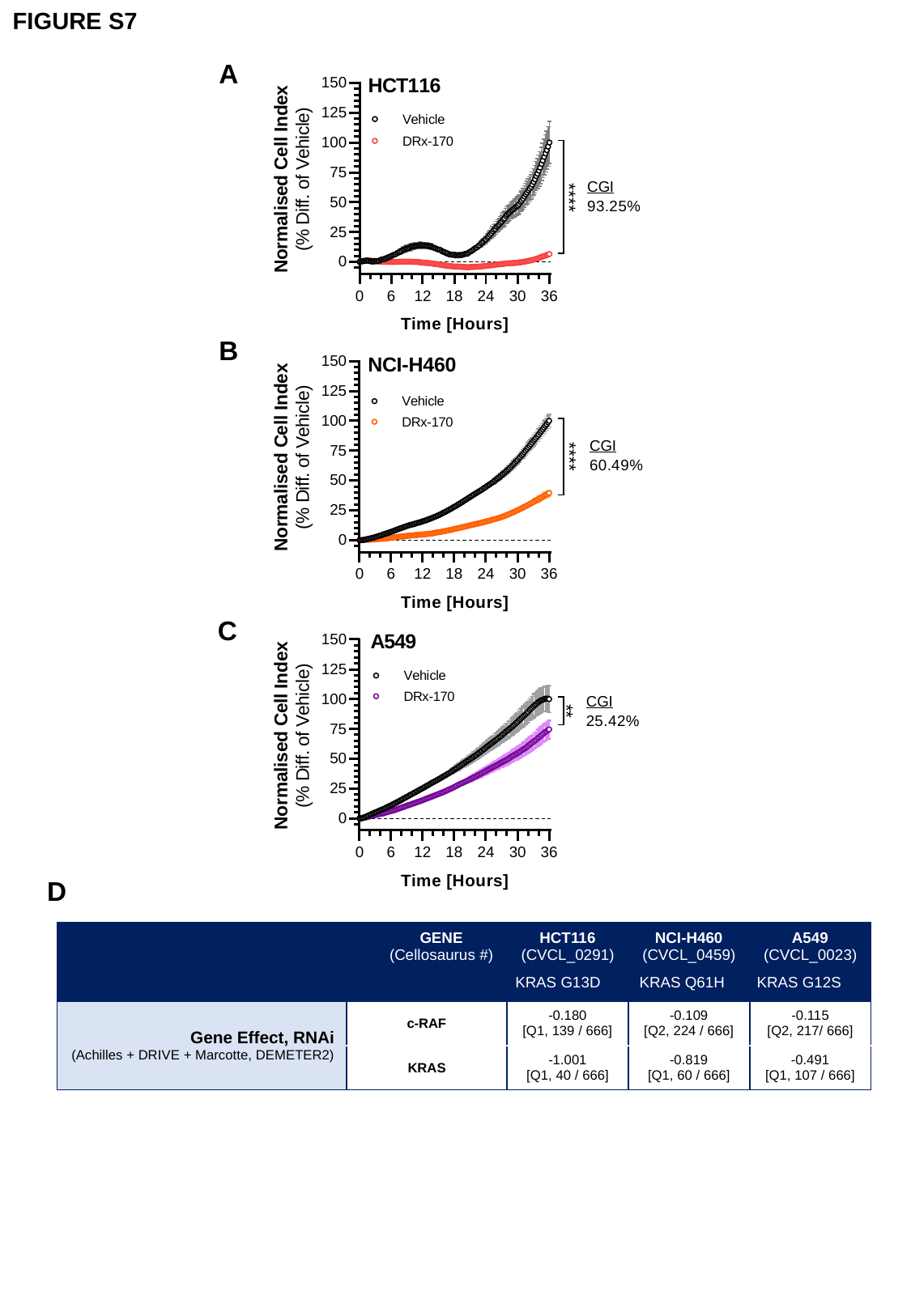

FIGURE S7
A
B
C
D
| | | | GENE (Cellosaurus #) | | HCT116 (CVCL\_0291) | NCI-H460 (CVCL\_0459) | A549 (CVCL\_0023) |
| --- | --- | --- | --- | --- | --- | --- | --- |
| | | Lineage | | | | | |
| Gene Effect, RNAi (Achilles + DRIVE + Marcotte, DEMETER2) | | c-RAF | | | -0.180 [Q1, 139 / 666] | -0.109 [Q2, 224 / 666] | -0.115 [Q2, 217/ 666] |
| | | KRAS | | | -1.001 [Q1, 40 / 666] | -0.819 [Q1, 60 / 666] | -0.491 [Q1, 107 / 666] |
KRAS G13D
KRAS Q61H
KRAS G12S

## Slide 8
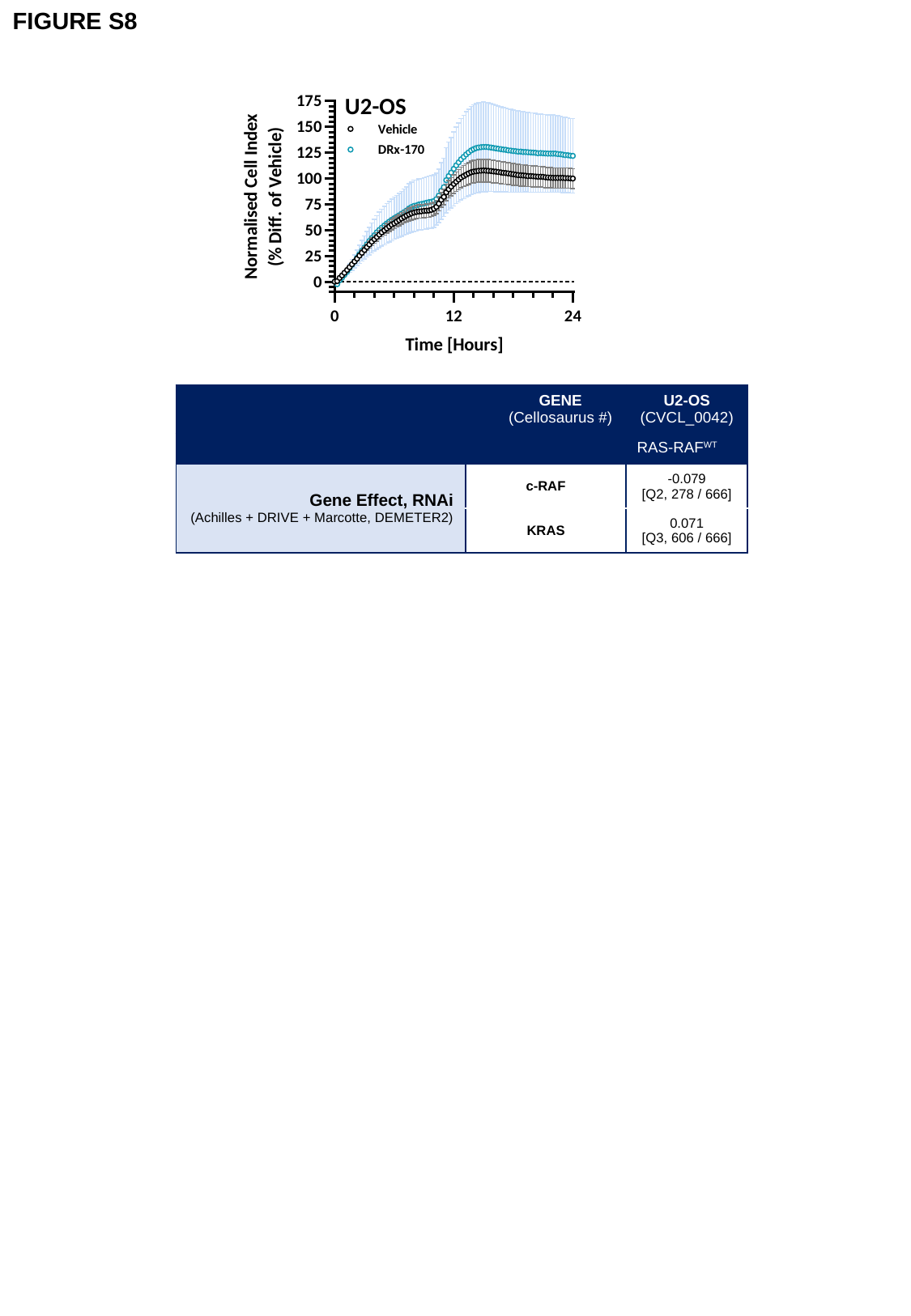

FIGURE S8
| | | GENE (Cellosaurus #) | U2-OS (CVCL\_0042) |
| --- | --- | --- | --- |
| | Lineage | | |
| Gene Effect, RNAi (Achilles + DRIVE + Marcotte, DEMETER2) | c-RAF | | -0.079 [Q2, 278 / 666] |
| | KRAS | | 0.071 [Q3, 606 / 666] |
RAS-RAFWT
